# Assessing the global dynamics of Nipah infection under vaccination and treatment: A novel computational modeling approach

**DOI:** 10.1371/journal.pone.0309360

**Published:** 2025-01-14

**Authors:** Fang Yu, Muhammad Younas Khan, Muhammad Bilal Riaz, Saif Ullah, Muhammad Farooq

**Affiliations:** 1 School of Mathematics and Data Sciences, Changji University, Changji, Xinjiang, China; 2 Department of Mathematics, University of Peshawar, Peshawar, Khyber Pakhtunkhwa, Pakistan; 3 IT4Innovations, VSB-Technical University of Ostrava, Ostrava, Czech Republic; 4 Department of Computer Science and Mathematics, Lebanese American University, Byblos, Lebanon; Kwame Nkrumah University of Science and Technology, GHANA

## Abstract

In biology and life sciences, fractal theory and fractional calculus have significant applications in simulating and understanding complex problems. In this paper, a compartmental model employing Caputo-type fractional and fractal-fractional operators is presented to analyze Nipah virus (NiV) dynamics and transmission. Initially, the model includes nine nonlinear ordinary differential equations that consider viral concentration, flying fox, and human populations simultaneously. The model is reconstructed using fractional calculus and fractal theory to better understand NiV transmission dynamics. We analyze the model’s existence and uniqueness in both contexts and instigate the equilibrium points. The clinical epidemiology of Bangladesh is used to estimate model parameters. The fractional model’s stability is examined using Ulam-Hyers and Ulam-Hyers-Rassias stabilities. Moreover, interpolation methods are used to construct computational techniques to simulate the NiV model in fractional and fractal-fractional cases. Simulations are performed to validate the stable behavior of the model for different fractal and fractional orders. The present findings will be beneficial in employing advanced computational approaches in modeling and control of NiV outbreaks.

## 1 Introduction

NiV an emerging and rapidly spreading zoonotic infection has a high mortality risk for its victims. The disease was initially reported in 1998 when an epidemic of encephalitis (inflammation of the brain) occurred in Singapore and Malaysia [1]. The virus is mostly transmitted to humans through fruit bats, also known as pigs, flying foxes and other domestic animals. Person-to-person transmission can also occur by direct contact with infected individuals’ bodily fluids, including as saliva, blood, or respiratory secretions. This infectious disease can cause mild to severe symptoms, such as headache, muscle discomfort, vomiting, fever, and respiratory illnesses. In severe cases, the infection might result in convulsions, encephalitis, or even coma [2]. Avoiding contagious animals and their products, as well as practicing good hygiene by washing hands frequently and cooking meat correctly, are major control tactics. Frequent outbreaks of this deadly infection significantly affected public health and the economy in countries like Bangladesh, India, and Malaysia [1, 3]. However, there is currently no effective vaccination or treatment for the NiV, which makes it a serious threat to both human and animal health due to its ability to cause severe respiratory infections [4].

Mathematical modeling stands for effective computational techniques for analyzing various dynamical features of transmissible diseases in a community. With the help of such models one can make decisions related to public health by offering valuable information on the dynamics of outbreaks and to set the most effective techniques for its intervention. In this regard, several transmission models with different mathematical structures have been investigated in the literature for example [5–7]. These models are commonly formulated using ordinary differential equations (ODEs), partial differential equations (PDEs) or stochastic systems [8–10]. Since ODE-based models can only reveal the disease’s temporal dynamics, they are commonly employed to investigate its spread in populations with high homogeneity. To account for the fact that disease transmission can vary in space, PDE-based models are used. The impact of small populations and minor occurrences can be captured using stochastic epidemic models, which incorporate randomness [11]. Several computational transmitting models have been developed to examine the intricate dynamics and efficacious control of NiV epidemics in various endemic regions. A deterministic model was studied in [12], which includes awareness and optimal treatment controls. In addition, they presented simulations showing how to control this infection and proved the existence theory for the control problem. In [13], the SEID-type compartmental model was used to study the effects of unprotected contact with dead NiV-infected bodies. A deterministic model-based study that examined several human-to-bat and pig transmission routes was published recently in [14]. In addition, the authors examined the model’s global dynamics and calculated its parameters using outbreak reported in Negeri Sembilan, Malaysia [14]. A nonlinear deterministic transmission model was used to examine the worldwide dynamics of NiV and potential prevention techniques in [15]. The NiV virus is widespread in several countries, including Bangladesh. In 2015, a NiV outbreak was discovered in Bangladesh. Mathematical models were given to investigate its dynamics and control [16, 17].

In fractional epidemic models, the classical integer-order derivatives used by traditional epidemic models are replaced by non-integer-order derivatives. The fractional model is enhanced with memory and hereditary effects preserved by fractional derivatives, which capture the intricate dynamical aspects seen in specific epidemics and enable a high level of problem accuracy [18–20]. One of the dependable and extensively utilized derivatives in the modeling technique is the Caputo-type derivative, which was presented in 1967 [21]. The Caputo-Fabrizio and Atangana-Baleanu operators are two more famous examples of fractional order derivatives [22, 23]. Novel fractal-fractional (FF) operations were developed by Atangana in 2017 [24], and they have created new opportunities for the examination of challenging problems, such as infectious diseases that display crossover behavior, through modeling methodologies. Fractal and fractional calculus, two fields with a long history of success, are used to derive these operators. Several phenomena, such as economic difficulties and the dynamic of COVID-19, have been studied using epidemic models built using (FF) operators [25]. In [26], the authors investigated the fundamental numerical and theoretical features of monkeypox infection by utilizing (FF) operators and the Caputo fractional. The dynamics and control of NiV were studied using computational transmission models with fractional derivatives, which were recently developed and are referenced in [27–29].

Recently, a novel computational ODE-based mathematical model addressing the NiV optimal control was developed in [30]. In the present study, we extend this model by incorporating fractional and FF modeling techniques. We develop a transmission model for NiV disease that accounts for its many dynamic characteristics. This approach allows for a more accurate representation of the disease’s transmission dynamics, considering long-term memory effects and complex dynamics that can not be captured by classical integer-order model. The following eight main parts make up this research. Basic definitions are covered in Section 2. A brief review of the procedure involved in formulating the classical NiV model with parameters estimation are covered in Section 3. Model formulation with a basic analysis of the Caputo NiV model of transmission is discussed in Section 4. The threshold value in relation to the model parameters is graphically analyzed in Section 5. The iterative solution and fractional model simulation are shown in Section 6. In Section 7, the model is extended with certain essential mathematical features in the form of an FF extension. Section 7 also details the NiV FF model’s numerical solution and simulation outcomes. The last conclusion are presented in Section 8.

## 2 Preliminaries

It is well-known in many fields, particularly in epidemiology, that advanced modeling methods based on FF operators are useful. We reviewed some key ideas about fractal and fractional calculus in this context [21, 24].

**Definition 1** *The following formula lists both the left and right Caputo-type derivatives of the function* Φ.

Dt0Ctς1Φ(t)=(Dt0t−(m−ς1)(ddt)mΦ(t))=1Γ(m-ς1)∫t0t((t-X)m-ς1-1Φm(X))dX,andDtCtς1Φ(t)=(DtT−(m−ς1)(-ddt)mΦ(t))=(-1)mΓ(m-ς1)∫tT((X-t)m-ς1-1Φm(X))dX. (1)

**Definition 2** *The following is the definition of the generalized version of a Mittag-Leffler function* $E_{r_{1},r_{2}}\left(x\right)$ *for real values x*:

$$\begin{array}{c} E_{r_{1},r_{2}}(x)=\sum_{m=0}^{\infty }\frac{x^{m}}{\Gamma (r_{1}m+r_{2})},\;\;\;r_{1},r_{2}>0, \end{array}$$ (2)

fulfills the below property:

$$\begin{array}{c} E_{r_{1},r_{2}}(x)=x\;E_{r_{1},r_{1}+r_{2}}(x)+\frac{1}{\Gamma (r_{2})}. \end{array}$$ (3)

The Laplace of $t^{r_{2}-1}E_{r_{1},r_{2}}(\pm \lambda t^{r_{1}})$ can be described as follows

$$\begin{array}{c} L[t^{r_{2}-1}E_{r_{1},r_{2}}(\pm \lambda t^{r_{1}})]=\frac{s^{r_{1}-r_{2}}}{s^{r_{1}}\mp \lambda }. \end{array}$$ (4)

**Definition 3** *A fractional system characterized by the Caputo-operator has a steady state represented by the following equation*:

DCtς1θ(t)=F(t,θ(t)),ς1∈(0,1), (5)

represents the point where *θ* = θ* and *F*(*t*, θ*) = 0 are satisfied.

**Definition 4** *The function* Φ, *quoted from* [24], *has the* *FF* *derivative as follows*.

DFF−P0,tς1,ς2(Φ)=1/(Γ(n-ς1))ddtς2∫0t(t-ς)n1-ς1-1Φ(ς)dς, (6)

*such that*, *ς*_1_, *ς*_2_ ∈ (*n*_1_ − 1, *n*_1_], *where* $n_{1}\in N$ *and* $\frac{d\Phi (\xi )}{d\xi^{\varsigma_{1}}}={\text{lim}}_{t\to \xi }\frac{\Phi -\Phi (\xi )}{t^{\varsigma_{1}}-\xi^{\varsigma_{2}}}$.

**Definition 5** *For* (6), *the* *FF* *integral is defined as follows*:

JFF−P0,tς1(Φ(t))=ς2Γ(ς1)∫0t(t-ς)ς1-1ςς2-1Φ(ς)dς. (7)

## 3 Modeling formulation approach

This section presents a brief explanation of the formulation of the NiV model using classical differential equations. The virus may be transferred via two approaches: food-borne transmission occurs when infected food is ingested, and direct transmission occurs between individuals through contact with both infected and deceased persons. The model has nine differential equations that represents the dynamic behavior of various populations. The state variable *V*(*t*) represents the overall rate of virus spreading by flying foxes at any given time *t*. The flying fox population is sub-divided into susceptible (*S*_*F*_) and infected (*I*_*F*_) groups. Flying foxes have been reported to be the natural reservoirs of the Nipah virus. The population of humans is classified into six compartments: humans who are susceptible (*S*_*H*_), vaccinated (*V*_*H*_), infected and can transmit infection (*I*_*H*_), treated (*T*_*H*_), recovered from infection (*R*_*H*_), and deceased infected humans (*D*_*H*_). Therefore: *N*_*F*_ = *S*_*F*_ + *I*_*F*_, *N*_*H*_ = *S*_*H*_ + *I*_*H*_ + *V*_*H*_ + *T*_*H*_ + *R*_*H*_. The sub-system demonstrating the dynamics of viral concentration and flying foxes is given by

$$\begin{array}{c} \begin{array}{c} V^{\prime }(t)=pI_{F}-\theta V,\\ S_{F}^{\prime }(t)=\Pi_{F}-\frac{\beta_{1}V}{N_{F}}S_{F}-d_{F}S_{F},\\ I_{F}^{\prime }(t)=\frac{\beta_{1}V}{N_{F}}S_{F}-d_{F}I_{F}. \end{array} \end{array}$$ (8)

In the above sub-model, the rate at which the virus sheds is represented by *p* and the rate at which it decays is denoted by *θ*. The population recruitment rate in the *S*_*F*_ compartment is represented by *Π*_*F*_, and individuals in this compartment die from natural causes at a rate of *d*_*F*_. The variable *β*_1_ represents the transmission rate from contaminated food to susceptible flying foxes. Mathematically, the force of infection is formulated by the formula $\frac{\beta_{1}VS_{F}}{N_{F}}$. The population in the susceptible class, denoted as *S*_*F*_, gets infected and moves to the infected class.

In this study, three modes of transmission are considered for the NiV: *β*_2_ for contaminated food, *β*_3_ for direct contact between infected individuals, and *β*_4_ for touching the bodies of infected individuals who have died, with a fraction *κ* indicating improper handling. By accounting for each of these three transmission rates, the force of infection may be determined.

$$\begin{array}{c} \lambda_{H}=\frac{\beta_{2}V+\beta_{3}I_{H}+\beta_{4}\kappa D_{H}}{N_{H}}. \end{array}$$ (9)

The sub-model that demonstrates the dynamics of humans is formulated as:

$$\begin{array}{c} \begin{array}{c} S_{H}^{\prime }(t)=\Pi_{H}-\frac{\left(\beta_{2}V+\beta_{3}I_{H}+\beta_{4}\kappa D_{H}\right)S_{H}}{N_{H}}-\left(d_{H}+\xi \right)S_{H}+\zeta V_{H}+\gamma R_{H},\\ V_{H}^{\prime }(t)=\xi S_{H}-\left(d_{H}+\zeta \right)V_{H},\\ I_{H}^{\prime }(t)=\frac{\left(\beta_{2}V+\beta_{3}I_{H}+\beta_{4}\kappa D_{H}\right)}{N_{H}}S_{H}-(\rho +d_{1}+\alpha_{1}+d_{H})I_{H},\\ T_{H}^{\prime }(t)=\rho I_{H}-(d_{H}+\alpha_{2})T_{H},\\ R_{H}^{\prime }(t)=\alpha_{1}I_{H}+\alpha_{2}T_{H}-(d_{H}+\gamma )R_{H},\\ D_{H}^{\prime }(t)=d_{1}I_{H}-\nu_{H}D_{H}. \end{array} \end{array}$$ (10)

In the sub-model (10), the human population is recruited at the rate Π_*H*_ and dies due to natural causes at the rate *d*_*H*_. The parameter *ξ* denotes the vaccination rate of susceptible humans, *γ* denotes the loss of immunity in the recovered class, *ζ* represents the loss of vaccine-induced immunity, and *ρ* is the treatment rate of infected humans. The recovery rates of infected and under-treatment humans are denoted by *α*_1_ and *α*_2_, respectively. The infection-induced death rate in the infected class is *d*_1_, and deceased humans who die due to NiV and are buried at the rate *ν*_*H*_.

The time behavior of the NiV can be examined by the following complete system of nonlinear differential equations, which combines sub-systems (8) and (10).

$$\begin{array}{c} \begin{array}{c} V^{\prime }(t)=pI_{F}-\theta V,\\ S_{F}^{\prime }(t)=\Pi_{F}-\frac{\beta_{1}V}{N_{F}}S_{F}-d_{F}S_{F},\\ I_{F}^{\prime }(t)=\frac{\beta_{1}V}{N_{F}}S_{F}-d_{F}I_{F},\\ S_{H}^{\prime }(t)=\Pi_{H}-\frac{\left(\beta_{2}V+\beta_{3}I_{H}+\beta_{4}\kappa D_{H}\right)S_{H}}{N_{H}}-\left(d_{H}+\xi \right)S_{H}+\zeta V_{H}+\gamma R_{H},\\ V_{H}^{\prime }(t)=\xi S_{H}-\left(d_{H}+\zeta \right)V_{H},\\ I_{H}^{\prime }(t)=\frac{\left(\beta_{2}V+\beta_{3}I_{H}+\beta_{4}\kappa D_{H}\right)}{N_{H}}S_{H}-(\rho +d_{1}+\alpha_{1}+d_{H})I_{H},\\ T_{H}^{\prime }(t)=\rho I_{H}-(d_{H}+\alpha_{2})T_{H},\\ R_{H}^{\prime }(t)=\alpha_{1}I_{H}+\alpha_{2}T_{H}-(d_{H}+\gamma )R_{H},\\ D_{H}^{\prime }(t)=d_{1}I_{H}-\nu_{H}D_{H}, \end{array} \end{array}$$ (11)

For the above system, the non-negative initial conditions (ICs) are

$$\begin{array}{c} \begin{array}{c} V\left(0\right)=V_{0}\geq 0,S_{F}\left(0\right)=S_{F0}\geq 0,I_{F}\left(0\right)=I_{F0}\geq 0,S_{H}\left(0\right)=S_{H0}\geq 0,V_{H}\left(0\right)=V_{H0}\geq 0,\\ I_{H}\left(0\right)=I_{H0}\geq 0,T_{H}\left(0\right)=T_{H0}\geq 0,R_{H}\left(0\right)=R_{H0}\geq 0,D_{H}\left(0\right)=D_{H0}\geq 0. \end{array} \end{array}$$ (12)

### 3.1 Parameter estimation of the model

In order to simulate the model, we estimate parameter values associated with outbreaks in Bangladesh. Two methods were used in this procedure. Bangladesh’s population has an average life expectancy of 73.57 years [31]. Consequently, the natural death rate is calculated to be $d_{H}=\frac{1}{73.57}$ annually. NiV infections in Bangladesh have a high case fatality rate, varying between 73% and 77%, and a recovery rate of 22.458% [32, 33]. As a result, the disease-induced death rate from Nipah infection is estimated to be roughly *d*_1_ = 0.760, and the recovery from infection is *α*_1_ = 0.22458 [32]. The relationship Π_*H*_ = *N*_*H*_(0) × *d*_*H*_, where *N*_*H*_(0) denotes Bangladesh’s cumulative population during 2015 [31, 34], is used to determine the human population recruitment rate Π_*H*_. Similarly, the literature mentioned in Table 1 is used to estimate the values of the remaining parameters. The detailed procedure can be found in [30].

**Table 1:** Physical description with estimated values of the model embedded parameters.

Parameter symbol	Physical description	value (per day)	Source
Π_*H*_	The rate of human recruitment	6295.17	[31]
Π_*F*_	The rate of Flying foxes recruitment	300.1	[44]
*d*_*H*_	The natural mortality rate of humans	$\frac{1}{73.57\times 365}$	[31]
*d*_*F*_	The natural mortality rate of Flying foxes	0.0250	[44]
*ν*	Rate of cremation for deceased people	0.50	[13]
*p*	The *I*_*F*_ virus production rate	0.147	fitted
*θ*	Decay rate of virus	0.0900	fitted
*γ*	Loss of immunity	0.850	[13]
*β*_1_	Transmission rate of *V* to *S*_*f*_ population	0.2910	fitted
*β*_2_	Transmission rate of *V* to humans	0.650	[44]
*β*_3_	Infection transmission rate due to *I*_*H*_	0.650	[13]
*β*_4_	Infection transmission rate due to *D*_*H*_	0.750	[13]
*α*_1_	Recovery rate of *I*_*H*_	0.2345	fitted
*α*_2_	Recovery rate from treated class	0.090	fitted
*d*_1_	Death rate caused by NiV	0.770	[45]
*κ*	Fraction of deceased not handled properly	0.001	[44]
*ρ*	Treatment rate	0.4	[45]
*ζ*	Vaccine waning rate	0.005	[30]
*ξ*	Vaccination rate for the susceptible persons	0.060	[30]

**Fig 1:**
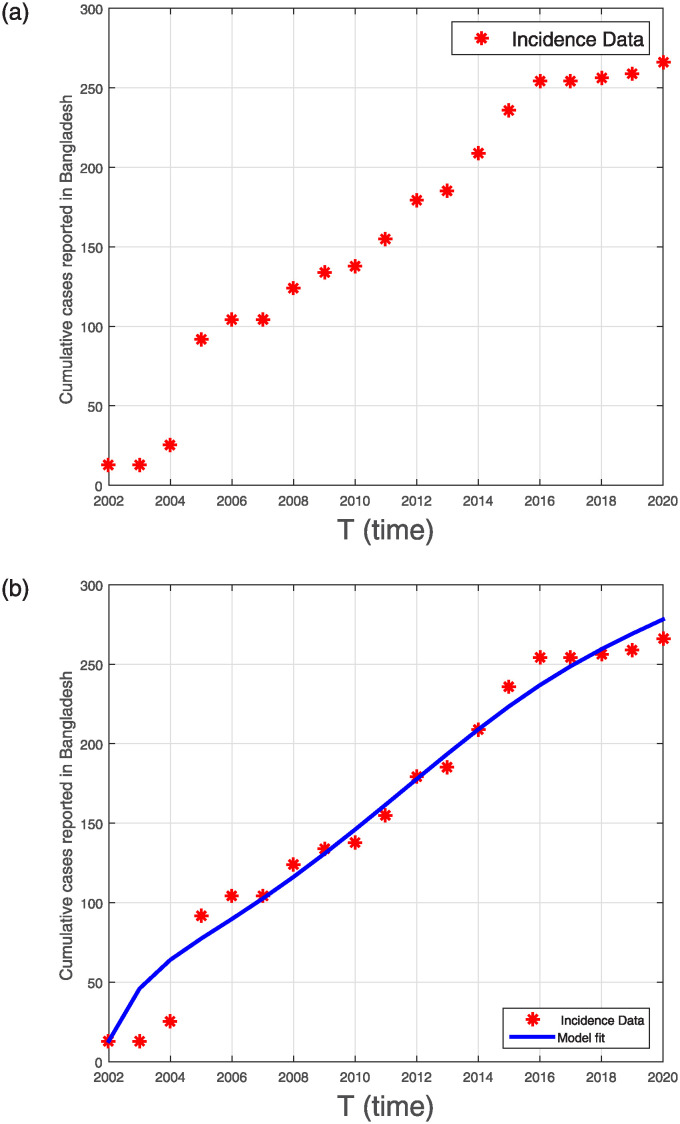
(a) Bangladesh’s cumulative confirmed cases reported from 2001-2020 (b) Fitting of the model.

We consider NiV-confirmed cases that were documented in Bangladesh during the 2001-2020 outbreaks to verify the model’s predictions. The documented cases of infection are taken from [2, 17]. Using a well-known statistical method called the least squares nonlinear regression minimization approach [35], we estimate the parameter values to minimize the differences (or residuals) between the actual observed data and the model predictions. The minimization is carried out using MATLAB, R2021b version, with the algorithm named “lsqcurvefit,” which relies on the following formula:

$$\hat{\Theta }=\text{arg}\;\text{min}\sum_{ȷ=1}^{n}{\left(H_{I_{t_{ȷ}}}-{\bar{H}}_{I_{t_{ȷ}}}\right)}^{2},$$

where *n* denotes the total number of data points, ${\bar{H}}_{I_{t_{ȷ}}}$ indicates the real data, and $H_{I_{t_{ȷ}}}$ represents the model-predicted cases at time *t*_*ȷ*_. Fig 1(a) shows the total number of NiV-related deaths (indicated by bold dots), and Fig 1(b) shows the simulated curve for the reported deaths (represented by a blue plot).

## 4 Formulation of the fractional NiV

This part extends the classical model (11) to the fractional case using the Caputo derivative. In certain real-world scenarios, many factors influence disease spread, particularly past infection rates, which affect current dynamics. Fractional order epidemic models provide a better representation of processes with long-term dependencies and allow for a deeper understanding of how interventions impact disease spread over time, considering the memory effect. These models can capture the complex nature of epidemics. Therefore, the integer-order derivative is replaced with the non-integer-order derivative in the NiV model (11). The Caputo fractional NiV epidemic model is comprised of the following system.

DCtς1V=pIF-θV,DCtς1SF=ΠF-β1VNFSF-dFSF,DCtς1IF=β1VNFSF-dFIF,DCtς1SH=ΠH-(β2V+β3IH+β4κDH)NHSH-c1SH+γRH+ζVH,DCtς1VH=ξSH-c2VH,DCtς1IH=(β2V+β3IH+β4κDH)NHSH-c3IH,DCtς1TH=ρIH-c4TH,DCtς1RH=α1IH+α2TH-c5RH,DCtς1DH=d1IH-νHDH, (13)

where, *c*_1_ = (*ξ* + *d*_*H*_), *c*_2_ = (*d*_*H*_ + *ζ*), *c*_3_ = (*ρ* + *d*_1_ + *α*_1_ + *d*_*H*_), *c*_4_ = (*α*_2_ + *d*_*H*_),*c*_5_ = (*γ* + *d*_*H*_) and subject to the ICs in (12).

### 4.1 Qualitative analysis of the fractional model

This section presents some of the fundamental characteristics of the fractional NiV epidemic model (13). The subsequent part presents the boundedness and invariant region for the model’s solution.

### 4.2 Invariantness and positivity

**Theorem 1** *The region* Θ = Θ_*F*_ × Θ_*H*_, *model* (13) *with the corresponding ICs* (12) *is positive invariant where*

$$\begin{array}{c} \begin{array}{c} \Theta_{F}=\{\;(V,\;S_{F},\;I_{F})\in \;R_{+}^{3}:N_{F}=S_{F}+I_{F}=\frac{\Pi_{F}}{d_{F}},V\leq \frac{p\Pi_{F}}{\theta d_{F}}\},\\ \Theta_{H}=\{(S_{H},\;I_{H},\;T_{H},\;R_{H},\;V_{H},\;D_{H})\in R_{+}^{6}:N_{H}=S_{H}+T_{H}+I_{H}+V_{H}+R_{H}\leq \frac{\Pi_{H}}{d_{H}},D_{H}\leq \frac{\Pi_{H}d_{1}}{\nu_{H}d_{H}}\}. \end{array} \end{array}$$ (14)

**Proof 1** *For model* (13), *we have the following*

DCtς1NF=CDtς1SF+CDtς1IF,=ΠF-dFNF,

*or*

DCtς1NF+dFNF=ΠF. (15)

*Make use of the Laplace transform*

L[DCtς1NF+dFNF]=L[ΠF],sς1NF(s)-sς1-1NF(0)+dFNF(s)=ΠFs,(sς1+dF)NF(s)=ΠFs+sς1-1NF(0),NF(s)=ΠFs(sς1+dF)+sς1-1NF(0)(sς1+dF).

*The utilization of inverse Laplace further proceeds as*

$$\begin{array}{c} N_{F}(t)\leq \frac{\Pi_{F}}{d_{F}}+(N_{F}\left(0\right)-\frac{\Pi_{F}}{d_{F}})E_{\varsigma_{1}}\left(-d_{F}t^{\varsigma_{1}}\right), \end{array}$$

*where Mittag-Leffler function of ς*_1_ *is defined by* $E_{\varsigma_{1}}\left(-d_{F}t^{\varsigma_{1}}\right)$ . *Furthermore, we derive that whenever t* → ∞, $N_{F}\left(t\right)\leq \frac{\Pi_{F}}{d_{F}}$, *if* $N_{F}\left(0\right)\leq \frac{\Pi_{F}}{d_{F}}$. *Consequently, for t* > 0, *solution* (13) *with ICs in* Θ_*F*_ *lies in* Θ_*F*_.

*In the following phase, consider the first equation in* (13) *and take the inequality into account* $I_{F}\leq \frac{\Pi_{F}}{d_{F}}$. *Consequently*,

DCtς1V(t)+θV≤pΠFdF,L[DCtς1V(t)+θV]≤L[pΠFdF],V(s)≤pΠFdFs(s1ς+θ)+sς1-1V(0)(s1ς+θ).

*Using a similar approach, we arrive at the following conclusion*:

$$\begin{array}{c} V\left(t\right)\leq \frac{p\Pi_{F}}{\theta d_{F}}+(V\left(0\right)-\frac{p\Pi_{F}}{\theta d_{F}})E_{\varsigma_{1}}\left(-\theta t^{\varsigma_{1}}\right). \end{array}$$

*Consequently, if the initial viral concentration, represented by* $V\left(0\right)\leq \frac{\Pi_{F}}{d_{F}}$ , *then as time approaches infinity*, $V\left(t\right)\leq \frac{p\Pi_{F}}{\theta d_{F}}$.

*For the human sub-population, we continue according to a similar approach as follows*.

$$\begin{array}{c} N_{H}(t)\leq \frac{\Pi_{H}}{d_{H}}+(N_{H}\left(0\right)-\frac{\Pi_{H}}{d_{H}})E_{\varsigma_{1}}\left(-d_{H}t^{\varsigma_{1}}\right), \end{array}$$

*and*

$$\begin{array}{c} D_{H}\left(t\right)\leq \frac{d_{H}}{\nu_{H}}\frac{\Pi_{H}}{d_{H}}+(D_{H}\left(0\right)-\frac{d_{1}}{\nu_{H}}\frac{\Pi_{H}}{d_{H}})E_{\varsigma_{1}}\left(-\nu_{H}t^{\varsigma_{1}}\right). \end{array}$$

*Thus, if* $N_{H}\left(0\right)\leq \frac{\Pi_{H}}{d_{H}}$ *and* $D_{H}\left(0\right)\leq \frac{d_{1}}{\nu_{H}}\frac{\Pi_{H}}{d_{H}}$ , *then* $N_{H}\left(t\right)\leq \frac{\Pi_{H}}{d_{H}}$ *and* $D_{H}\left(t\right)\leq \frac{d_{1}}{\nu_{H}}\frac{\Pi_{H}}{d_{H}}$ (*as t* ≥ 0 *and t* → ∞). *Therefore, for the system solution* (13), *the aforementioned region maintains the feature of positive invariantness*.

### 4.3 The existence and the uniqueness

This section aims to show the existence and positiveness of the solution of the system (13). We achieve this using the generalized mean value theorem described in [36].

**Theorem 2** *The NiV model* (13) *in the Caputo framework possesses a non-negative and unique solution*.

**Proof 2** *In order to determine the desired result, we rely on the information provided in reference* [37]. *The solution’s existence can be easily confirmed utilizing the previously mentioned procedure. Furthermore, by utilizing Remark 3.2 from* [37], *we can prove the solution’s uniqueness. To verify that the solution is non-negative, it is essential to ensure that the vector field points towards the positive orthant of* $R_{+}^{9}$ *on every hyperplane that defines it. Based on* Eq (13), *it can be derived that*:

DCtς1V(t)|V=0=pIF≥0,DCtς1SF(t)|SF=0=ΠF≥0,DCtς1IF(t)|IF=0=λFSF≥0,DCtς1SH(t)|SH=0=ΠH+γRH+ζVH≥0,DCtς1VH(t)|VH=0=ξSH≥0,DCtς1IH(t)|IH=0=λHSH≥0,DCtς1TH(t)|TH=0=ρIH≥0,DCtς1RH(t)|RH=0=α1IH+α2TH≥0,DCtς1DH(t)|DH=0=d1IH≥0.

*Based on the findings discussed in the aforementioned literature, it can be inferred that the solutions will exist in the positive nine-dimensional real space, denoted as* $R_{+}^{9}$ , *for all t* ≥ 0.

### 4.4 The equilibria and the threshold number $R_{0}$

The model (13) has three equilibria. The trivial or Nipah virus-free equilibrium (NVFE) is shown as below:

$$\begin{array}{c} E^{0}=(V^{0},S_{F}^{0},I_{F}^{0},S_{H}^{0},V_{H}^{0},I_{H}^{0},T_{H}^{0},R_{H}^{0},D_{H}^{0})=(0,\frac{\Pi_{F}}{d_{F}},0,\frac{c_{2}\Pi_{H}}{c_{1}c_{2}-\zeta \xi },\frac{\xi \Pi_{H}}{c_{1}c_{2}-\zeta \xi },0,0,0,0). \end{array}$$ (16)

The threshold number $R_{0}$ is calculated through the use of a well-known next-generation technique [30, 38]. The following equation shows $R_{0}$ as a result.

$$\begin{array}{c} R_{0}=\text{max}\{R_{F}^{0},R_{H}^{0}\}=\text{max}\{R_{F}^{0},R_{h_{1}}^{0}+R_{h_{2}}^{0}\}=\text{max}\{\frac{p\beta_{1}}{\theta d_{F}},\frac{\beta_{3}c_{2}}{c_{3}\left(c_{2}+\xi \right)}+\frac{\beta_{4}c_{2}d_{1}\kappa }{\nu_{H}c_{3}\left(c_{2}+\xi \right)}\}, \end{array}$$ (17)

### 4.5 Infected flying fox-free equilibrium point

**Theorem 3** *The model* (13) *has a unique infected flying fox-free equilibrium* (*IFFE*) *whenever*, $R_{H}^{0}>1$.

**Proof 3** *For the virus and human compartments, we can solve equations* (13) *simultaneously to get an equation for* $S_{F}=\frac{\Pi_{F}}{d_{F}}$, *I*_*F*_ = 0, *and V* = 0, *considering the* (*IFFE*) *point*.

$$\begin{array}{c} E^{*}=(0,\frac{\Pi_{F}}{d_{F}},0,\;S_{H}^{*},\;I_{H}^{*},\;T_{H}^{*},\;V_{H}^{*},\;R_{H}^{*},\;D_{H}^{*}), \end{array}$$ (18)

*where*

$$\begin{array}{c} \begin{array}{c} S_{H}^{*}=\frac{c_{2}c_{3}c_{4}c_{5}\Pi_{H}}{c_{3}c_{4}c_{5}\left(c_{1}c_{2}-\zeta \xi \right)+c_{2}\lambda_{H}\left(c_{3}c_{4}c_{5}-\gamma \left(\alpha_{1}c_{4}+\alpha_{2}\rho \right)\right)},\\ V_{H}^{*}=\frac{\xi }{c_{2}}S_{H}^{*},\\ I_{H}^{*}=\frac{1}{c_{3}}\lambda_{H}^{*}S_{H}^{*},\\ T_{H}^{*}=\frac{\rho }{c_{3}c_{4}}\lambda_{H}^{*}S_{H}^{*},\\ R_{H}^{*}=\frac{\left(\alpha_{1}c_{4}+\alpha_{2}\rho \right)}{c_{3}c_{4}c_{5}}\lambda_{H}^{*}S_{H}^{*},\\ D_{H}^{*}=\frac{d_{1}}{\nu_{H}c_{3}}\lambda_{H}^{*}S_{H}^{*}, \end{array} \end{array}$$ (19)

*where*,

$$\begin{array}{c} \lambda_{H}^{*}=\frac{\beta_{2}V^{*}+\beta_{3}I_{H}^{*}+\beta_{4}\kappa D_{H}^{*}}{N_{H}^{*}}. \end{array}$$ (20)

*Further, putting* (19) *in* (20), *we obtain*

$$\begin{array}{c} \varrho_{1}\lambda_{H}^{*}+\varrho_{2}=0, \end{array}$$ (21)

*where the coefficients are*

$\varrho_{1}=\nu_{H}c_{3}c_{4}c_{5}\left(\xi +c_{2}\right)\Pi_{H}(1-R_{H}^{0}),$

*ϱ*_2_ = *c*_2_*ν*_*H*_Π_*H*_(*α*_2_ *ρ* + *α*_1_*c*_4_ + *c*_5_(*c*_4_+ *ρ*)).

*Thus, there exists a unique* (*IFFE*) *point if* $R_{H}^{0}>1$.

#### 4.5.1 Endemic equilibrium state of the NiV

System (13) can be solved simultaneously at steady state to get the following results:

$$\begin{array}{c} E^{**}=(V^{**},\;S_{F}^{**},\;I_{F}^{**},\;S_{H}^{**},\;I_{H}^{**},\;T_{H}^{**},\;V_{H}^{**},\;R_{H}^{**},\;D_{H}^{**}), \end{array}$$ (22)

where,

$$\begin{array}{c} \begin{array}{c} V^{**}=\frac{\Pi_{F}^{**}(p\beta_{1}-\theta d_{F})}{\beta_{1}\theta d_{F}},\\ S_{F}^{**}=\frac{\theta \Pi_{F}^{**}}{p\beta_{1}},\\ I_{F}^{**}=\frac{\Pi_{F}^{**}(p\beta_{1}-\theta d_{F})}{p\beta_{1}d_{F}},\\ S_{H}^{**}=\frac{c_{2}c_{3}c_{4}c_{5}\Pi_{H}}{c_{3}c_{4}c_{5}\left(c_{1}c_{2}-\zeta \xi \right)+c_{2}\lambda_{H}\left(c_{3}c_{4}c_{5}-\gamma \left(\alpha_{1}c_{4}+\alpha_{2}\rho \right)\right)},\\ V_{H}^{**}=\frac{\xi }{c_{2}}S_{H}^{**},\\ I_{H}^{**}=\frac{1}{c_{3}}\lambda_{H}^{**}S_{H}^{**},\\ T_{H}^{**}=\frac{\rho }{c_{3}c_{4}}\lambda_{H}^{**}S_{H}^{**},\\ R_{H}^{**}=\frac{\left(\alpha_{1}c_{4}+\alpha_{2}\rho \right)}{c_{3}c_{4}c_{5}}\lambda_{H}^{**}S_{H}^{**},\\ D_{H}^{**}=\frac{d_{1}}{\nu_{H}c_{3}}\lambda_{H}^{**}S_{H}^{**}, \end{array} \end{array}$$ (23)

where,

$$\begin{array}{c} \lambda_{H}^{**}=\frac{\beta_{2}V^{**}+\beta_{3}I_{H}^{**}+\beta_{4}\kappa D_{H}^{**}}{N_{H}^{**}}. \end{array}$$ (24)

Further, putting (23) in (24), we obtain

$$\begin{array}{c} \varpi_{0}\lambda_{H}^{**2}+\varpi_{1}\lambda_{H}^{**}+\varpi_{2}=0, \end{array}$$ (25)

where the coefficients are

$\varpi_{2}=\nu_{H}\beta_{2}c_{3}c_{4}c_{5}\Pi_{F}\left(c_{1}c_{2}-\zeta \xi \right)\left(1-R_{F}^{0}\right),$

$\varpi_{1}=\nu_{H}\beta_{2}c_{2}\Pi_{F}\left(1-R_{F}^{0}\right)\left[c_{3}c_{4}c_{5}-\gamma (\alpha_{2}\rho +\alpha_{1}c_{4})\right]+\nu_{H}c_{3}c_{4}c_{5}\beta_{1}\Pi_{H}\left(\xi +c_{2}\right)\left(1-R_{H}^{0}\right)$

*ϖ*_0_ = *β*_1_*c*_2_Π_*H*_[(*α*_1_*c*_4_ + *α*_2_*ρ*) + *c*_5_(*c*_4_ + *ρ*)].

Therefore, the below theorem is achieved.

**Theorem 4** *(i) If* $\varpi_{2}<0\Leftrightarrow R_{F}\geq 1$ , *the NVEE point* $E^{**}$ *exists and will be unique*.

*(ii) If* $\left(\varpi_{1}<0\wedge \varpi_{2}=0\right)$ ∨ $\varpi_{1}^{2}-4\varpi_{0}\varpi_{2}=0$, *the point* $E^{**}$ *will be unique*.

*(iii) If* *ϖ*_1_ < 0, *ϖ*_2_ > 0 *and the discriminant is positive, then the model has two NVEE*.

*(iv) An NVEE cannot be seen anywhere else*.

Based on condition (i), the model has a unique *NVEE*.

#### 4.5.2 Ulam-Hyers stability of the non-integer NiV model

Stabilities investigate the resilience of solutions of non-integer and integer differential systems under small perturbations to their initial conditions. The stability analysis of fractional systems is typically performed using the widely accepted Ulam-Hyers-Rassias (UHR) and Ulam-Hyers (UH) criteria, which were first presented in [39, 40]. These stabilities criteria are utilized to manage the efficacy of the model, specifically in situations where obtaining exact solutions may be challenging.

By applying the stability conditions known as UH and UHR, we will prove the stable result of a Caputo-NiV model (13) and present a similar concept from [41, 42].

**Definition 6** *The NiV epidemic system* (13) *is* *UH*-*stable if there exist* $\;0<D_{G_{i}}\in R,i=1,\cdots ,9$ *so that* ∀ *δ*_*i*_ > 0 and ∀ $(V^{**},S_{F}^{**},I_{F}^{**},S_{H}^{**},I_{H}^{**},T_{H}^{**},V_{H}^{**},R_{H}^{**},D_{H}^{**})\in Y$ *and satisfies*

$$\begin{array}{c} \left\{ \begin{array}{c} {|}^{C}D_{t}^{\varsigma_{1}}V^{**}\left(t\right)-K_{1}\left(V^{**}\left(t\right),S_{F}^{**}\left(t\right),I_{F}^{**}\left(t\right),S_{H}^{**}\left(t\right),V_{H}^{**}\left(t\right),I_{H}^{**}\left(t\right),T_{H}^{**}\left(t\right),R_{H}^{**}\left(t\right),D_{H}^{**}\left(t\right)\right)|<\delta_{1},\\ {|}^{C}D_{t}^{\varsigma_{1}}S_{F}^{**}\left(t\right)-K_{2}\left(V^{**}\left(t\right),S_{F}^{**}\left(t\right),I_{F}^{**}\left(t\right),S_{H}^{**}\left(t\right),V_{H}^{**}\left(t\right),I_{H}^{**}\left(t\right),T_{H}^{**}\left(t\right),R_{H}^{**}\left(t\right),D_{H}^{**}\left(t\right)\right)|<\delta_{2},\\ {|}^{C}D_{t}^{\varsigma_{1}}I_{F}^{**}\left(t\right)-K_{3}\left(V^{**}\left(t\right),S_{F}^{**}\left(t\right),I_{F}^{**}\left(t\right),S_{H}^{**}\left(t\right),V_{H}^{**}\left(t\right),I_{H}^{**}\left(t\right),T_{H}^{**}\left(t\right),R_{H}^{**}\left(t\right),D_{H}^{**}\left(t\right)\right)|<\delta_{3},\\ {|}^{C}D_{t}^{\varsigma_{1}}S_{H}^{**}\left(t\right)-K_{4}\left(V^{**}\left(t\right),S_{F}^{**}\left(t\right),I_{F}^{**}\left(t\right),S_{H}^{**}\left(t\right),V_{H}^{**}\left(t\right),I_{H}^{**}\left(t\right),T_{H}^{**}\left(t\right),R_{H}^{**}\left(t\right),D_{H}^{**}\left(t\right)\right)|<\delta_{4},\\ {|}^{C}D_{t}^{\varsigma_{1}}V_{H}^{**}\left(t\right)-K_{5}\left(V^{**}\left(t\right),S_{F}^{**}\left(t\right),I_{F}^{**}\left(t\right),S_{H}^{**}\left(t\right),V_{H}^{**}\left(t\right),I_{H}^{**}\left(t\right),T_{H}^{**}\left(t\right),R_{H}^{**}\left(t\right),D_{H}^{**}\left(t\right)\right)|<\delta_{5},\\ {|}^{C}D_{t}^{\varsigma_{1}}I_{H}^{**}\left(t\right)-K_{6}\left(V^{**}\left(t\right),S_{F}^{**}\left(t\right),I_{F}^{**}\left(t\right),S_{H}^{**}\left(t\right),V_{H}^{**}\left(t\right),I_{H}^{**}\left(t\right),T_{H}^{**}\left(t\right),R_{H}^{**}\left(t\right),D_{H}^{**}\left(t\right)\right)|<\delta_{6},\\ {|}^{C}D_{t}^{\varsigma_{1}}T_{H}^{**}\left(t\right)-K_{7}\left(V^{**}\left(t\right),S_{F}^{**}\left(t\right),I_{F}^{**}\left(t\right),S_{H}^{**}\left(t\right),V_{H}^{**}\left(t\right),I_{H}^{**}\left(t\right),T_{H}^{**}\left(t\right),R_{H}^{**}\left(t\right),D_{H}^{**}\left(t\right)\right)|<\delta_{7},\\ {|}^{C}D_{t}^{\varsigma_{1}}R_{H}^{**}\left(t\right)-K_{8}\left(V^{**}\left(t\right),S_{F}^{**}\left(t\right),I_{F}^{**}\left(t\right),S_{H}^{**}\left(t\right),V_{H}^{**}\left(t\right),I_{H}^{**}\left(t\right),T_{H}^{**}\left(t\right),R_{H}^{**}\left(t\right),D_{H}^{**}\left(t\right)\right)|<\delta_{8},\\ {|}^{C}D_{t}^{\varsigma_{1}}D_{H}^{**}\left(t\right)-K_{9}\left(V^{**}\left(t\right),S_{F}^{**}\left(t\right),I_{F}^{**}\left(t\right),S_{H}^{**}\left(t\right),V_{H}^{**}\left(t\right),I_{H}^{**}\left(t\right),T_{H}^{**}\left(t\right),R_{H}^{**}\left(t\right),D_{H}^{**}\left(t\right)\right)|<\delta_{9}, \end{array}\right. \end{array}$$ (26)

∃ $(V,S_{F},I_{F},S_{H},I_{H},T_{H},V_{H},R_{H},D_{H})\in Y$ *following* (13) *with*

$$\begin{array}{c} \left\{ \begin{array}{c} |V^{**}\left(t\right)-V\left(t\right)|\leq D_{G_{1}}\delta_{1},\\ |S_{F}^{**}\left(t\right)-S_{F}\left(t\right)|\leq D_{G_{2}}\delta_{2},\\ |I_{F}^{**}\left(t\right)-I_{F}\left(t\right)|\leq D_{G_{3}}\delta_{3},\\ |S_{H}^{**}\left(t\right)-S_{H}\left(t\right)|\leq D_{G_{4}}\delta_{4},\\ |V_{H}^{**}\left(t\right)-V_{H}\left(t\right)|\leq D_{G_{5}}\delta_{5},\\ |I_{H}^{**}\left(t\right)-I_{H}\left(t\right)|\leq D_{G_{6}}\delta_{6},\\ |T_{H}^{**}\left(t\right)-T_{H}\left(t\right)|\leq D_{G_{7}}\delta_{7},\\ |R_{H}^{**}\left(t\right)-R_{H}\left(t\right)|\leq D_{G_{8}}\delta_{8},\\ |D_{H}^{**}\left(t\right)-D_{H}\left(t\right)|\leq D_{G_{9}}\delta_{9}. \end{array}\right. \end{array}$$ (27)

**Remark 1** $(V^{**},\;S_{F}^{**},\;I_{F}^{**},\;S_{F}^{**},\;S_{H}^{**},\;I_{H}^{**},\;T_{H}^{**},\;V_{H}^{**},\;R_{H}^{**},\;D_{H}^{**})\in Y$ *is a solution the NiV fractional model* (13) *iff* $\exists \;{\text{u}}_{i}\in C\left(\left[0,T\right],R\right)$ *such that for all*, *t in* T, *we reached to the conditions stated below*

*(i)*. $|{\text{u}}_{i}\left(t\right)|<\delta_{i},\;\left(i=1,\cdots ,9\right)$, *and*

(ii).{DCtς1V**(t)=K1(V**(t),SF**(t),IF**(t),SH**(t),IH**(t),VH**(t),TH**(t),RH**(t),DH**(t))+u1(t),DCtς1SF**(t)=K2(V**(t),SF**(t),IF**(t),SH**(t),IH**(t),VH**(t),TH**(t),RH**(t),DH**(t))+u2(t),DCtς1IF**(t)=K3(V**(t),SF**(t),IF**(t),SH**(t),IH**(t),VH**(t),TH**(t),RH**(t),DH**(t))+u3(t),DCtς1SH**(t)=K4(V**(t),SF**(t),IF**(t),SH**(t),IH**(t),VH**(t),TH**(t),RH**(t),DH**(t))+u4(t),DCtς1VH**(t)=K5(V**(t),SF**(t),IF**(t),SH**(t),IH**(t),VH**(t),TH**(t),RH**(t),DH**(t))+u5(t),DCtς1IH**(t)=K6(V**(t),SF**(t),IF**(t),SH**(t),IH**(t),VH**(t),TH**(t),RH**(t),DH**(t))+u6(t),DCtς1TH**(t)=K7(V**(t),SF**(t),IF**(t),SH**(t),IH**(t),VH**(t),TH**(t),RH**(t),DH**(t))+u7(t),DCtς1RH**(t)=K8(V**(t),SF**(t),IF**(t),SH**(t),IH**(t),VH**(t),TH**(t),RH**(t),DH**(t))+u8(t),DCtς1DH**(t)=K9(V**(t),SF**(t),IF**(t),SH**(t),IH**(t),VH**(t),TH**(t),RH**(t),DH**(t))+u9(t). (28)

**Definition 7** *Concerning the mentioned functions, the NiV epidemic model* (13) *is* *UHR* *stable*. *χ*_*i*_, *i* = 1 ⋯, 9 *whenever there exists* $\;0<D_{G_{i},\varrho_{i}\left(t\right)}\in R,i=1,\cdots ,9$ *such that* ∀ *δ*_*i*_ > 0 *and* ∀ $(V^{**},S_{F}^{**},I_{F}^{**},S_{H}^{**},I_{H}^{**},T_{H}^{**},V_{H}^{**},R_{H}^{**},D_{H}^{**})\in Y$ *and satisfies*

$$\begin{array}{c} \left\{ \begin{array}{c} {|}^{C}D_{t}^{\varsigma_{1}}V^{**}\left(t\right)-K_{1}\left(V^{**}\left(t\right),S_{F}^{**}\left(t\right),I_{F}^{**}\left(t\right),S_{H}^{**}\left(t\right),I_{H}^{**}\left(t\right),V_{H}^{**}\left(t\right),T_{H}^{**}\left(t\right),R_{H}^{**}\left(t\right),D_{H}^{**}\left(t\right)\right)|<\delta_{1}\varrho_{1}\left(t\right),\\ {|}^{C}D_{t}^{\varsigma_{1}}S_{F}^{**}\left(t\right)-K_{2}\left(V^{**}\left(t\right),S_{F}^{**}\left(t\right),I_{F}^{**}\left(t\right),S_{H}^{**}\left(t\right),I_{H}^{**}\left(t\right),V_{H}^{**}\left(t\right),T_{H}^{**}\left(t\right),R_{H}^{**}\left(t\right),D_{H}^{**}\left(t\right)\right)|<\delta_{2}\varrho_{2}\left(t\right),\\ {|}^{C}D_{t}^{\varsigma_{1}}I_{F}^{**}\left(t\right)-K_{3}\left(V^{**}\left(t\right),S_{F}^{**}\left(t\right),I_{F}^{**}\left(t\right),S_{H}^{**}\left(t\right),I_{H}^{**}\left(t\right),V_{H}^{**}\left(t\right),T_{H}^{**}\left(t\right),R_{H}^{**}\left(t\right),D_{H}^{**}\left(t\right)\right)|<\delta_{3}\varrho_{3}\left(t\right),\\ {|}^{C}D_{t}^{\varsigma_{1}}S_{H}^{**}\left(t\right)-K_{4}\left(V^{**}\left(t\right),S_{F}^{**}\left(t\right),I_{F}^{**}\left(t\right),S_{H}^{**}\left(t\right),I_{H}^{**}\left(t\right),V_{H}^{**}\left(t\right),T_{H}^{**}\left(t\right),R_{H}^{**}\left(t\right),D_{H}^{**}\left(t\right)\right)|<\delta_{4}\varrho_{4}\left(t\right),\\ {|}^{C}D_{t}^{\varsigma_{1}}V_{H}^{**}\left(t\right)-K_{5}\left(V^{**}\left(t\right),S_{F}^{**}\left(t\right),I_{F}^{**}\left(t\right),S_{H}^{**}\left(t\right),I_{H}^{**}\left(t\right),V_{H}^{**}\left(t\right),T_{H}^{**}\left(t\right),R_{H}^{**}\left(t\right),D_{H}^{**}\left(t\right)\right)|<\delta_{5}\varrho_{5}\left(t\right),\\ {|}^{C}D_{t}^{\varsigma_{1}}I_{H}^{**}\left(t\right)-K_{6}\left(V^{**}\left(t\right),S_{F}^{**}\left(t\right),I_{F}^{**}\left(t\right),S_{H}^{**}\left(t\right),I_{H}^{**}\left(t\right),V_{H}^{**}\left(t\right),T_{H}^{**}\left(t\right),R_{H}^{**}\left(t\right),D_{H}^{**}\left(t\right)\right)|<\delta_{6}\varrho_{6}\left(t\right),\\ {|}^{C}D_{t}^{\varsigma_{1}}T_{H}^{**}\left(t\right)-K_{7}\left(V^{**}\left(t\right),S_{F}^{**}\left(t\right),I_{F}^{**}\left(t\right),S_{H}^{**}\left(t\right),I_{H}^{**}\left(t\right),V_{H}^{**}\left(t\right),T_{H}^{**}\left(t\right),R_{H}^{**}\left(t\right),D_{H}^{**}\left(t\right)\right)|<\delta_{7}\varrho_{7}\left(t\right),\\ {|}^{C}D_{t}^{\varsigma_{1}}R_{H}^{**}\left(t\right)-K_{8}\left(V^{**}\left(t\right),S_{F}^{**}\left(t\right),I_{F}^{**}\left(t\right),S_{H}^{**}\left(t\right),I_{H}^{**}\left(t\right),V_{H}^{**}\left(t\right),T_{H}^{**}\left(t\right),R_{H}^{**}\left(t\right),D_{H}^{**}\left(t\right)\right)|<\delta_{8}\varrho_{8}\left(t\right),\\ {|}^{C}D_{t}^{\varsigma_{1}}D_{H}^{**}\left(t\right)-K_{9}\left(V^{**}\left(t\right),S_{F}^{**}\left(t\right),I_{F}^{**}\left(t\right),S_{H}^{**}\left(t\right),I_{H}^{**}\left(t\right),V_{H}^{**}\left(t\right),T_{H}^{**}\left(t\right),R_{H}^{**}\left(t\right),D_{H}^{**}\left(t\right)\right)|<\delta_{9}\varrho_{9}\left(t\right), \end{array}\right. \end{array}$$ (29)

*there exists* $(V,S_{F},I_{F},S_{H},I_{H},T_{H},V_{H},R_{H},D_{H})\in Y$ *taking into account the NiV epidemic model* (13) *with*

$$\begin{array}{c} \left\{ \begin{array}{c} |V^{**}\left(t\right)-V\left(t\right)|\leq D_{G_{1}}\delta_{1}\varrho_{1}\left(t\right),\\ |S_{F}^{**}\left(t\right)-S_{F}\left(t\right)|\leq D_{G_{2}}\delta_{2}\varrho_{2}\left(t\right),\\ |I_{F}^{**}\left(t\right)-I_{F}\left(t\right)|\leq D_{G_{3}}\delta_{3}\varrho_{3}\left(t\right),\\ |S_{H}^{**}\left(t\right)-S_{H}\left(t\right)|\leq D_{G_{4}}\delta_{4}\varrho_{4}\left(t\right),\\ |V_{H}^{**}\left(t\right)-V_{H}\left(t\right)|\leq D_{G_{5}}\delta_{5}\varrho_{5}\left(t\right),\\ |I_{H}^{**}\left(t\right)-I_{H}\left(t\right)|\leq D_{G_{6}}\delta_{6}\varrho_{6}\left(t\right),\\ |T_{H}^{**}\left(t\right)-T_{H}\left(t\right)|\leq D_{G_{7}}\delta_{7}\varrho_{7}\left(t\right),\\ |R_{H}^{**}\left(t\right)-R_{H}\left(t\right)|\leq D_{G_{8}}\delta_{8}\varrho_{8}\left(t\right),\\ |D_{H}^{**}\left(t\right)-D_{H}\left(t\right)|\leq D_{G_{9}}\delta_{9}\varrho_{9}\left(t\right). \end{array}\right. \end{array}$$ (30)

**Remark 2** $(V^{**},S_{F}^{**},I_{F}^{**},S_{H}^{**},V_{H}^{**},I_{H}^{**},T_{H}^{**},R_{H}^{**},D_{H}^{**})\in Y$ *is a solution of the Caputo system* (13) *iff* ∃ ${\text{u}}_{i}\in C\left(\left[0,T\right],R\right),$ *based on* $V^{**},I_{F}^{**},S_{F}^{**},S_{H}^{**},I_{H}^{**},T_{H}^{**},V_{H}^{**},R_{H}^{**},$ *and* $D_{H}^{**}$, *correspondingly, such that* ∀ t∈T:

*(i)*. $|{\text{u}}_{i}\left(t\right)|<\varrho_{i}\left(t\right)\delta_{i},\;\left(i=1,\cdots ,9\right)$, *and*

(ii).{DCtς1V**(t)=K1(V**(t),SF**(t),IF**(t),SH**(t),IH**(t),VH**(t),TH**(t),RH**(t),DH**(t))+u1(t),DCtς1SF**(t)=K2(V**(t),SF**(t),IF**(t),SH**(t),IH**(t),VH**(t),TH**(t),RH**(t),DH**(t))+u2(t),DCtς1IF**(t)=K3(V**(t),SF**(t),IF**(t),SH**(t),IH**(t),VH**(t),TH**(t),RH**(t),DH**(t))+u3(t),DCtς1SH**(t)=K4(V**(t),SF**(t),IF**(t),SH**(t),IH**(t),VH**(t),TH**(t),RH**(t),DH**(t))+u4(t),DCtς1VH**(t)=K5(V**(t),SF**(t),IF**(t),SH**(t),IH**(t),VH**(t),TH**(t),RH**(t),DH**(t))+u5(t),DCtς1IH**(t)=K6(V**(t),SF**(t),IF**(t),SH**(t),IH**(t),VH**(t),TH**(t),RH**(t),DH**(t))+u6(t),DCtς1TH**(t)=K7(V**(t),SF**(t),IF**(t),SH**(t),IH**(t),VH**(t),TH**(t),RH**(t),DH**(t))+u7(t),DCtς1RH**(t)=K8(V**(t),SF**(t),IF**(t),SH**(t),IH**(t),VH**(t),TH**(t),RH**(t),DH**(t))+u8(t),DCtς1DH**(t)=K9(V**(t),SF**(t),IF**(t),SH**(t),IH**(t),VH**(t),TH**(t),RH**(t),DH**(t))+u9(t). (31)

**Theorem 5** *On* T≔[0,T] , *the Caputo NiV epidemic model* (13) *is* *UH* *stable. so that*

$$\hat{\Delta }{\hat{\Phi }}_{i}<1,\;\;\;\;i\in \left\{1,\cdots ,9\right\},$$

*where* ${\hat{\Phi }}_{i}$ *and* $\hat{\Delta }$ *are given as*

$$\begin{array}{c} \;{\hat{\Phi }}_{i}:\;\;\;{\hat{\Phi }}_{1}=\theta ,\;\;{\hat{\Phi }}_{2}=\beta_{1}\Delta_{1}+d_{F},\;\;{\hat{\Phi }}_{3}=d_{F},{\hat{\Phi }}_{4}=\beta_{2}\Delta_{2}+\beta_{3}\Delta_{3}+\beta_{4}\kappa \Delta_{4}+c_{1},{\hat{\Phi }}_{5}=c_{2},\\ {\hat{\Phi }}_{6}=c_{3},{\hat{\Phi }}_{7}=c_{4},{\hat{\Phi }}_{8}=c_{5},\;\;{\hat{\Phi }}_{9}=\nu_{H}, \end{array}$$

*(A1) gives the following outcomes, if the condition (A1) is true*:

$$\|\frac{V}{N_{F}}\|\leq \Delta_{1},\|\frac{V}{N_{H}}\|\leq \Delta_{2},\|\frac{I_{H}}{N_{H}}\|\leq \Delta_{3},\;\|\frac{D_{H}}{N_{H}}\|\leq \Delta_{4}.$$

**Proof 4** *Let* *δ*_2_ *be a positive real number and* $S_{F}^{**}\in Y$ *so that*

$${|}^{C}D_{t}^{\varsigma_{1}}S_{F}^{**}\left(t\right)-K_{2}\left(V^{**}\left(t\right),S_{F}^{**}\left(t\right),I_{F}^{**}\left(t\right),S_{H}^{**}\left(t\right),V_{H}^{**}\left(t\right),I_{H}^{**}\left(t\right),T_{H}^{**}\left(t\right),R_{H}^{**}\left(t\right),D_{H}^{**}\left(t\right)\right)|<\delta_{2}.$$

*Then, using the result presented in Remark 1, there exists a* ${\text{u}}_{1}\left(t\right)$ *such that*

DCtς1SF**(t)=K2(V**(t),SF**(t),IF**(t),SH**(t),VH**(t),IH**(t),TH**(t),RH**(t),DH**(t))+u2(t),

*and* $|{\text{u}}_{1}\left(t\right)|\leq \delta_{1}$ . *Thus*,

$$\begin{array}{cc} S_{F}^{**}\left(t\right) & ={S_{F}}_{0}+\frac{1}{\Gamma (\varsigma_{1})}\int_{0}^{t}{\left(t-r\right)}^{\varsigma_{1}-1}K_{2}\left(V^{**}\left(r\right),S_{F}^{**}\left(r\right),I_{F}^{**}\left(r\right),S_{H}^{**}\left(r\right),V_{H}^{**}\left(r\right),I_{H}^{**}\left(r\right),T_{H}^{**}\left(r\right),R_{H}^{**}\left(r\right),D_{H}^{**}\left(r\right)\right)\text{d}r\\ & +\frac{1}{\Gamma (\varsigma_{1})}\int_{0}^{t}{\left(t-r\right)}^{\varsigma_{1}-1}{\text{u}}_{2}\left(r\right)\text{d}r. \end{array}$$

*According to the uniqueness theorem, we have a unique solution* $S_{F}\in Y$ *for the NiV non-integer system* (13). *The equation that represents* $S_{F}\left(t\right)$ *is as follows*:

$$\begin{array}{c} S_{F}\left(t\right)={S_{F}}_{0}+\frac{1}{\Gamma (\varsigma_{1})}\int_{0}^{t}{\left(t-r\right)}^{\varsigma_{1}-1}K_{2}\left(V^{**}\left(r\right),S_{F}^{**}\left(r\right),I_{F}^{**}\left(r\right),S_{H}^{**}\left(r\right),V_{H}^{**}\left(r\right),I_{H}^{**}\left(r\right),T_{H}^{**}\left(r\right),R_{H}^{**}\left(r\right),D_{H}^{**}\left(r\right)\right)\text{d}r. \end{array}$$

*Then*,

$$\begin{array}{cc} |S_{F}^{**}\left(t\right)-S_{F}\left(t\right)| & \leq \frac{1}{\Gamma (\varsigma_{1})}\int_{0}^{t}{\left(t-r\right)}^{\varsigma_{1}-1}\left|{\text{u}}_{1}\left(r\right)\right|\text{d}r\\ & +\frac{1}{\Gamma (\varsigma_{1})}\int_{0}^{t}{\left(t-r\right)}^{\varsigma_{1}-1}\\ & \times |K_{2}\left(V^{**}\left(r\right),S_{F}^{**}\left(r\right),I_{F}^{**}\left(r\right),S_{H}^{**}\left(r\right),V_{H}^{**}\left(r\right),I_{H}^{**}\left(r\right),T_{H}^{**}\left(r\right),R_{H}^{**}\left(r\right),D_{H}^{**}\left(r\right)\right)\\ & -K_{2}\left(V\left(r\right),S_{F}\left(r\right),I_{F}\left(r\right),S_{H}\left(r\right),V_{H}\left(r\right),I_{H}\left(r\right),T_{H}\left(r\right),R_{H}\left(r\right),D_{H}\left(r\right)\right)|\text{d}r\\ & \leq \hat{\Delta }\delta_{2}+\hat{\Delta }{\hat{\Phi }}_{2}\left\|S_{F}^{**}-S_{F}\right\|. \end{array}$$

*By considering the supremum norm of the inequality mentioned above, we can conclude*,

$$\|S_{F}^{**}-S_{F}\|-\hat{\Delta }{\hat{\Phi }}_{2}\left\|S_{F}^{**}-S_{F}\right\|\leq \hat{\Delta }\delta_{2}.$$

*Thus*,

$$\|S_{F}^{**}-S_{F}\|\leq \frac{\hat{\Delta }\delta_{2}}{1-\hat{\Delta }{\hat{\Phi }}_{2}}.$$

*If* $D_{Q_{2}}=\frac{\hat{\Delta }}{1-\hat{\Delta }{\hat{\Phi }}_{2}}$ , *therefore*, $\|S_{F}^{**}-S_{F}\|\leq D_{Q_{2}}\delta_{2}$. *In a similar approach*,

$$\|V^{**}-V\|\leq D_{Q_{1}}\delta_{1},\;\;\;\;\|I_{F}^{**}-I_{F}\|\leq D_{Q_{3}}\delta_{3},\;\;\;\;\left\|S_{H}^{**}-S_{H}\right\|\leq D_{Q_{4}}\delta_{4},$$

$$\|V_{H}^{**}-V_{H}\|\leq D_{Q_{5}}\delta_{5},\;\;\;\;\left\|I_{H}^{**}-I_{H}\right\|\leq D_{Q_{6}}\delta_{6},$$

$$\|T_{H}^{**}-T_{H}\|\leq D_{Q_{7}}\delta_{7},\;\;\;\;\|R_{H}^{**}-R_{H}\|\leq D_{Q_{8}}\delta_{8},\;\;\;\;\left\|D_{H}^{**}-D_{H}\right\|\leq D_{Q_{9}}\delta_{9},$$

*where*,

$$D_{Q_{i}}=\frac{\hat{\Delta }}{1-\hat{\Delta }{\hat{\Phi }}_{i}},\;\;\;\;\left(i\in \left\{1\cdots ,9\right\}\right).$$

*This leads to the derivation of* (13) *is* *UH* *stable*.

**Theorem 6** *Suppose* (*A*’), ∋, ∃ *a non-decreasing mappings* $\Psi_{i}\in C\left(\left[0,T\right],R^{+}\right),(i\in \left\{1,\cdots ,9\right\}$) *and* ∃ $\Xi_{\Psi_{i}}>0$ *if* ∀ t∈T,

IC0,tτΨi(t)<ΞΨiΨi(t),i∈{1,⋯,9}. (32)

*Then fractional NiV epidemic model* (13) *holds the* *UHR*-*stability if* (*A*1) *fulfills*.

**Proof 5** *Let* $S_{F}^{**}\in Y$ *and a positive real number* *δ*_2_ *so that*

$${|}^{C}D_{t}^{\varsigma_{1}}S_{F}^{**}\left(t\right)-K_{2}\left(V^{**}\left(t\right),S_{F}^{**}\left(t\right),I_{F}^{**}\left(t\right),S_{H}^{**}\left(t\right),V_{H}^{**}\left(t\right),I_{H}^{**}\left(t\right),T_{H}^{**}\left(t\right),R_{H}^{**}\left(t\right),D_{H}^{**}\left(t\right)\right)|<\delta_{2}\Psi_{2}\left(t\right),$$

*By applying Remark 2 result*, ${\text{u}}_{2}\left(t\right)$ *exists such that*

DCtς1SF**(t)=K2(V**(t),SF**(t),IF**(t),SH**(t),VH**(t),IH**(t),TH**(t),RH**(t),DH**(t))+u2(t),

*and* $|{\text{u}}_{2}\left(t\right)|\leq \delta_{2}\Psi_{2}\left(t\right)$. *Thus*,

$$\begin{array}{cc} S_{F}^{**}\left(t\right) & ={S_{F}}_{0}+\frac{1}{\Gamma (\varsigma_{1})}\int_{0}^{t}{\left(t-r\right)}^{\varsigma_{1}-1}K_{2}\left(V^{**}\left(r\right),S_{F}^{**}\left(r\right),I_{F}^{**}\left(r\right),S_{H}^{**}\left(r\right),V_{H}^{**}\left(r\right),I_{H}^{**}\left(r\right),T_{H}^{**}\left(r\right),R_{H}^{**}\left(r\right),D_{H}^{**}\left(r\right)\right)\text{d}r\\ & +\frac{1}{\Gamma (\varsigma_{1})}\int_{0}^{t}{\left(t-r\right)}^{\varsigma_{1}-1}{\text{u}}_{2}\left(r\right)\text{d}r. \end{array}$$

*We assume that* $S_{F}\in Y$ *is a unique solution of* (13) *based on the uniqueness theorem*. *Afterwards*, $S_{F}\left(t\right)$ *can be shown as*:

$$\begin{array}{c} S_{F}\left(t\right)={S_{F}}_{0}+\frac{1}{\Gamma (\varsigma_{1})}\int_{0}^{t}{\left(t-r\right)}^{\varsigma_{1}-1}K_{2}\left(V^{**}\left(r\right),S_{F}^{**}\left(r\right),I_{F}^{**}\left(r\right),S_{H}^{**}\left(r\right),V_{H}^{**}\left(r\right),I_{H}^{**}\left(r\right),T_{H}^{**}\left(r\right),R_{H}^{**}\left(r\right),D_{H}^{**}\left(r\right)\right)\text{d}r. \end{array}$$

*Then*,

$$\begin{array}{cc} |S_{F}^{**}\left(t\right)-S_{F}\left(t\right)| & \leq \frac{1}{\Gamma (\varsigma_{1})}\int_{0}^{t}{\left(t-r\right)}^{\varsigma_{1}-1}\left|{\text{u}}_{1}\left(r\right)\right|\text{d}r+\frac{1}{\Gamma (\varsigma_{1})}\int_{0}^{t}{\left(t-r\right)}^{\varsigma_{1}-1}\\ & \times |K_{2}\left(V^{**}\left(r\right),S_{F}^{**}\left(r\right),I_{F}^{**}\left(r\right),S_{H}^{**}\left(r\right),V_{H}^{**}\left(r\right),I_{H}^{**}\left(r\right),T_{H}^{**}\left(r\right),R_{H}^{**}\left(r\right),D_{H}^{**}\left(r\right)\right)\\ & -K_{2}\left(V\left(r\right),S_{F}\left(r\right),I_{F}\left(r\right),S_{H}\left(r\right),V_{H}\left(r\right),I_{H}\left(r\right),T_{H}\left(r\right),R_{H}\left(r\right),D_{H}\left(r\right)\right)|\text{d}r\\ & \leq \hat{\Delta }\Xi_{\Psi_{2}}\delta_{2}\left(t\right)+\hat{\Delta }{\hat{\Phi }}_{2}\left\|S_{F}^{**}-S_{F}\right\|. \end{array}$$

*Taking supremum norm over both sides, we arrived to the subsequent results*

$$\|S_{F}^{**}-S_{F}\|-\hat{\Delta }{\hat{\Phi }}_{2}\left\|S_{F}^{**}-S_{F}\right\|\leq \hat{\Delta }\delta_{2}.$$

*Thus*,

$$\|S_{F}^{**}-S_{F}\|\leq \frac{\hat{\Delta }\Xi_{\Psi_{2}}\delta_{2}\left(t\right)}{1-\hat{\Delta }{\hat{\Phi }}_{2}}.$$

*If* $D_{Q_{2},\Phi_{2}}=\frac{\Xi_{\Psi_{2}}}{1-\hat{\Delta }{\hat{\Phi }}_{2}}$ , *then* $\|S_{F}^{**}-S_{F}\|\leq D_{Q_{2},\Phi_{2}}\delta_{2}\Phi_{2}\left(t\right)$. *In a similar approach*,

$$\|V^{**}-V\|\leq D_{Q_{1},\Phi_{1}}\delta_{1}\Phi_{1}\left(t\right),\;\;\;\;\|I_{F}^{**}-I_{F}\|\leq D_{Q_{2},\Phi_{3}}\delta_{3}\Phi_{3}\left(t\right),\;\;\;\;\left\|S_{H}^{**}-S_{H}\right\|\leq D_{Q_{4},\Phi_{4}}\delta_{4}\Phi_{4}\left(t\right),$$

$$\|V_{H}^{**}-V_{H}\|\leq D_{Q_{5},\Phi_{5}}\delta_{5}\Phi_{5}\left(t\right),\;\;\;\;\left\|I_{H}^{**}-I_{H}\right\|\leq D_{Q_{6},\Phi_{6}}\delta_{6}\Phi_{6}\left(t\right),$$

$$\|T_{H}^{**}-T_{H}\|\leq D_{Q_{7},\Phi_{7}}\delta_{7}\Phi_{7}\left(t\right),\;\;\;\;\|R_{H}^{**}-R_{H}\|\leq D_{Q_{8},\Phi_{8}}\delta_{8}\Phi_{8}\left(t\right),\;\;\;\;\left\|D_{H}^{**}-D_{H}\right\|\leq D_{Q_{9},\Phi_{9}}\delta_{9}\Phi_{9}\left(t\right),$$

*where*,

$$D_{Q_{i},\Phi_{i}}=\frac{\Xi_{\Psi_{i}}}{1-\hat{\Delta }{\hat{\Phi }}_{i}},\;\;\;\;\left(i\in \left\{1\cdots ,9\right\}\right).$$

*Therefore, the* *UHR*-*stability for the Caputo fractional NiV system* (13) *is obtained*.

## 5 Interpretations of $R_{H}^{0}$ versus model parameters

**Fig 2:**
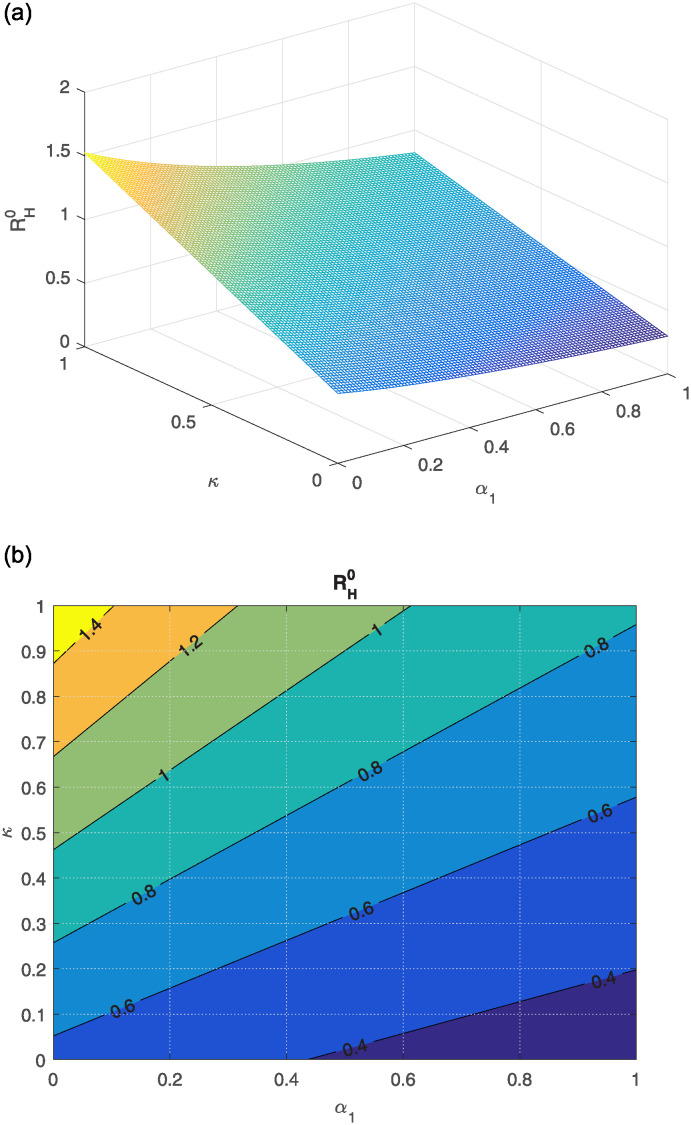
The plot shows the effect of *κ* (the unsafe rate of transportation of corpses resulting in NiV transmission) and *α*_1_ (rate of recovery of infectious humans) on $R_{H}^{0}$, along with the corresponding contour plot.

**Fig 3:**
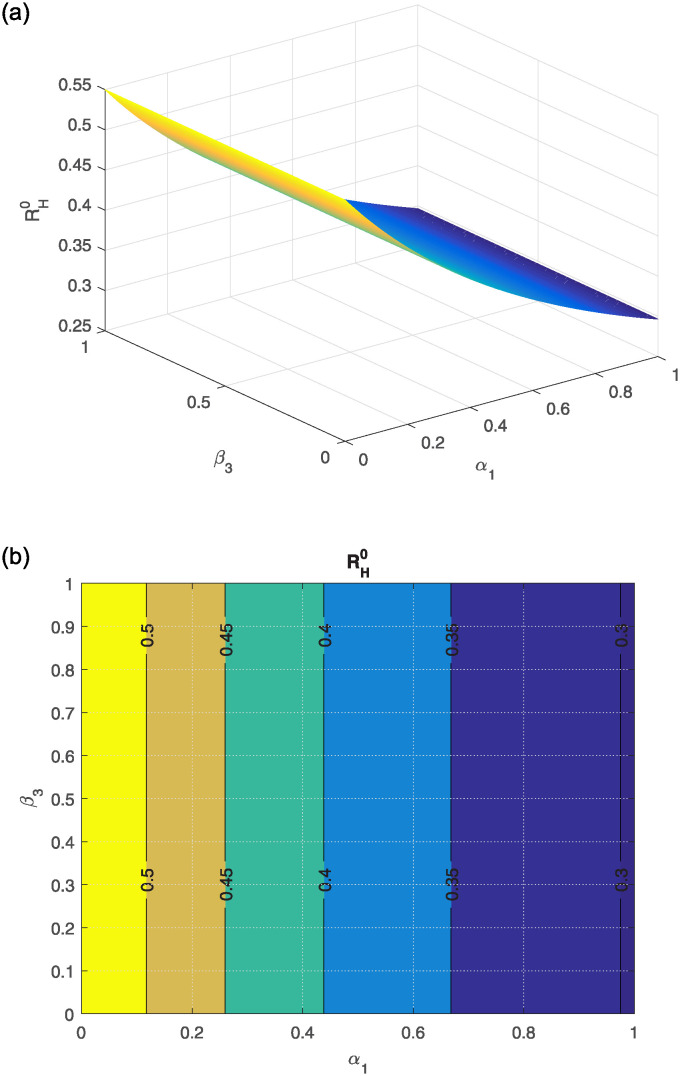
(a) shows the effect of *β*_3_ (Nipah rate of infection transmission relative to infectious human) and *α*_1_ (rate of recovery) on $R_{H}^{0}$, (b) the corresponding contour plot respectively.

**Fig 4:**
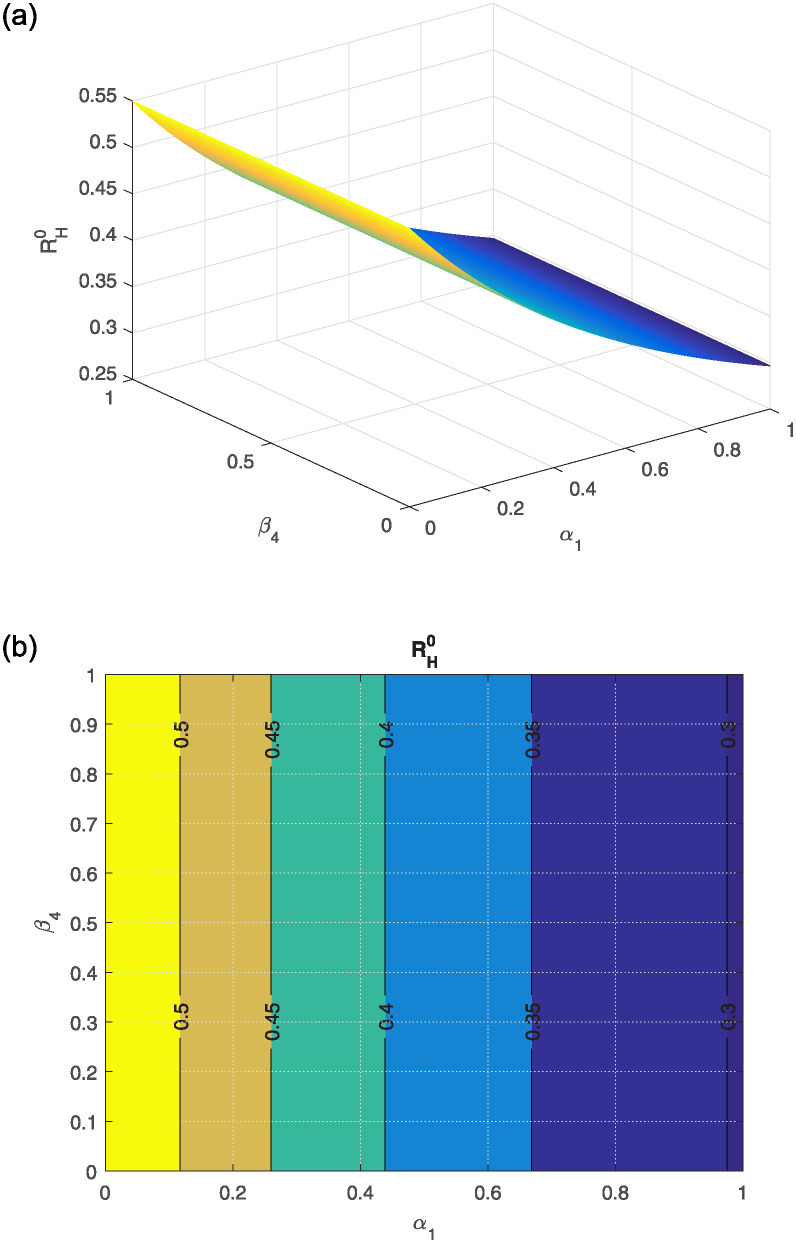
(a) shows the effect of *β*_4_ (rate of infection transmission relative to infectious human) and *α*_1_ (rate of recovery) on $R_{H}^{0}$, (b) the corresponding contour plot.

**Fig 5:**
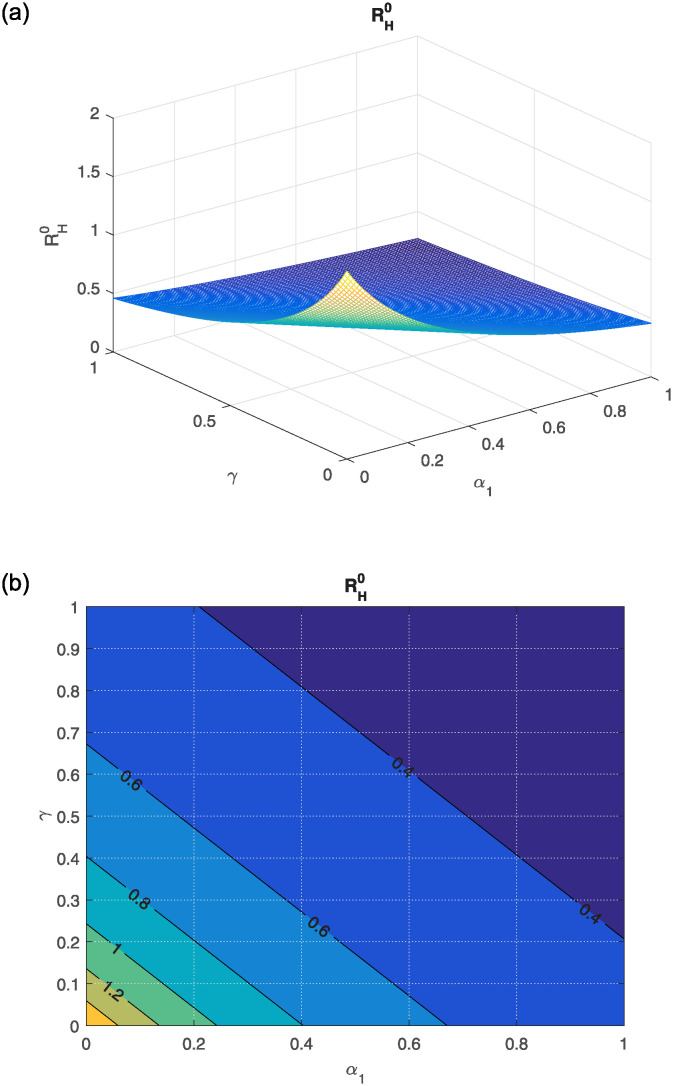
(a) Influence of *γ* (loss of immunity) and *α*_1_ (rate of recovery) on $R_{H}^{0}$, (b) the corresponding contour plot.

**Fig 6:**
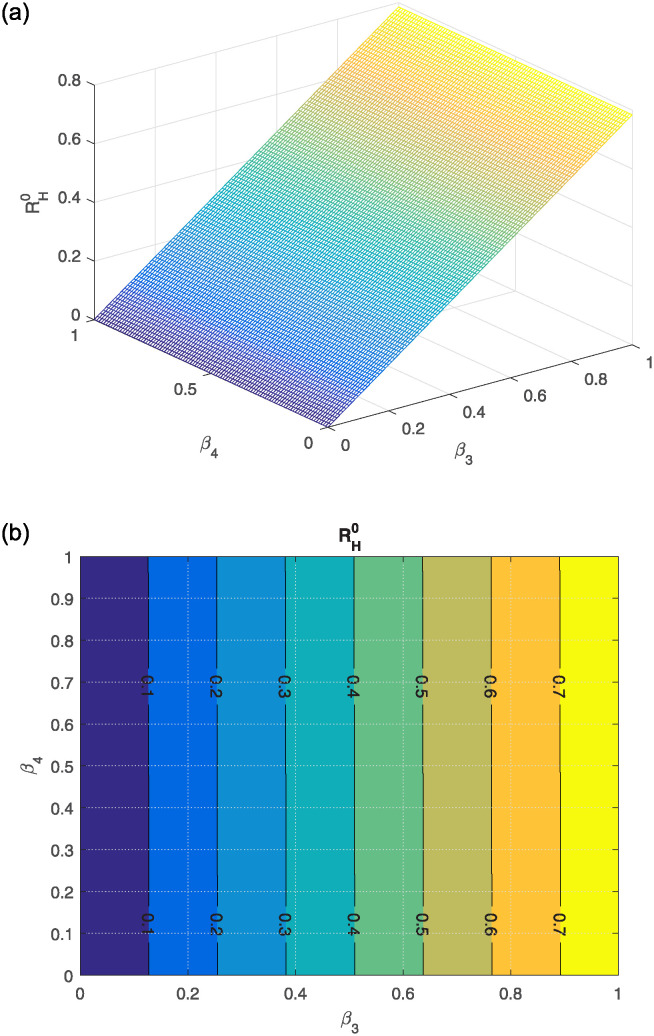
(a) shows how *β*_4_ (rate of transmission compared to NiV-positive dead people) and *β*_3_ (transmission rate relative to infectious humans) affect $R_{H}^{0}$, (b) the corresponding contour plot.

The effect of different model parameters on the threshold number $R_{H}^{0}$ is examined in this section. The contour plots corresponding to the most influential parameters are graphically interpreted in Figs 2–6. The impact of *κ* (indicating the rate of unsafe corpse transportation contributing to the NiV spreading) and *α*_1_ (rate of recovery of infected individuals) on $R_{H}^{0}$ is shown in Fig 2. It can be seen that as *α*_1_ rises and *κ* falls, the value of $R_{H}^{0}$ decreases to less than unity. The combined effects of transmission rates from deceased NiV-positive humans *β*_4_, transmission rates from infectious humans *β*_3_, and recovery rates *α*_1_ on $R_{H}^{0}$ are shown in Figs 3 and 4. We found that the value of $R_{H}^{0}$ can be significantly decreased by raising the recovery rate *α*_1_ and decreasing the disease transmission rates *β*_3_ and *β*_4_. With a decline in disease transmission rates *β*_3_ and *β*_4_, and an increase in the rate of recovery *α*_1_, the value of $R_{H}^{0}$ decreases dramatically. The effect of the immunity loss rate *γ* and the recovery rate *α*_1_ on $R_{H}^{0}$ is depicted in Fig 5. As can be observed, decreasing the loss of immunity rate *γ* and raising the recovery rate *α*_1_ can both aid in lowering the value of $R_{H}^{0}$. Finally, Fig 6 illustrates how changes in the transmission rates *β*_3_ and *β*_4_ affect the behavior of $R_{H}^{0}$. An outbreak may be possible if these parameters increase to the point where $R_{H}^{0}$ exceeds unity.

## 6 Computational analysis of the model

The iterative solution of the NiV Cputo model is investigated using the fractional Euler’s scheme [43]. The NiV compartmental model in Caputo case (13) is designed as follows:

{DCtς1h(t)=Q(t,h(t)),h(0)=h0,0<t<∞. (33)

In (33), $h\left(t\right)=\left(V,\;S_{F},\;I_{F},\;S_{H},\;I_{H},\;T_{H},\;V_{H},\;R_{H},\;D_{H}\right)\in R^{9}$ and *h*_0_ indicates the respective initial vector. Further,

$$\begin{array}{cc} Q(t,h(t)) & =(Q_{1}\left(t,h\left(t\right)\right),Q_{2}\left(t,h\left(t\right)\right),Q_{3}\left(t,h\left(t\right)\right),Q_{4}\left(t,h\left(t\right)\right),Q_{5}\left(t,h\left(t\right)\right),Q_{6}\left(t,h\left(t\right)\right)\\ & ,Q_{7}\left(t,h\left(t\right)\right)),Q_{8}\left(t,h\left(t\right)\right),Q_{9}\left(t,h\left(t\right)\right) \end{array}$$

denotes a real-valued vector function and satisfies the Lipschitz condition, where

$$\begin{array}{c} \left\{ \begin{array}{cc} Q_{1}\left(t,h\left(t\right)\right) & =pI_{F}-\theta V,\\ Q_{2}\left(t,h\left(t\right)\right) & =\Pi_{F}-\frac{\beta_{1}V}{N_{F}}S_{F}-d_{F}S_{F},\\ Q_{3}\left(t,h\left(t\right)\right) & =\frac{\beta_{1}V}{N_{F}}S_{F}-d_{F}I_{F},\\ Q_{4}\left(t,h\left(t\right)\right) & =\Pi_{H}-\frac{\left(\beta_{2}V+\beta_{3}I_{H}+\beta_{4}\kappa D_{H}\right)}{N_{H}}S_{H}-c_{1}S_{H}+\gamma R_{H}+\zeta V_{H},\\ Q_{5}\left(t,h\left(t\right)\right) & =\xi S_{H}-c_{2}V_{H},\\ Q_{6}\left(t,h\left(t\right)\right) & =\frac{\left(\beta_{2}V+\beta_{3}I_{H}+\beta_{4}\kappa D_{H}\right)}{N_{H}}S_{H}-c_{3}I_{H},\\ Q_{7}\left(t,h\left(t\right)\right) & =\rho I_{H}-c_{4}T_{H},\\ Q_{8}\left(t,h\left(t\right)\right) & =\alpha_{1}I_{H}+\alpha_{2}T_{H}-c_{5}R_{H},\\ Q_{9}\left(t,h\left(t\right)\right) & =d_{1}I_{H}-\nu_{H}D_{H}. \end{array}\right. \end{array}$$ (34)

From the problem (33) after applying the Caputo integral we deduced

$$\begin{array}{c} h\left(t\right)=h_{0}+\frac{1}{\Gamma (\varsigma_{1})}\int_{0}^{t}{\left(t-\varsigma \right)}^{\varsigma_{1}-1}Q\left(\varsigma ,h\left(\varsigma \right)\right)d\varsigma . \end{array}$$ (35)

A uniform grid is used to split the interval [0, T] with the step-size is $\hbar =\frac{T-0}{m}$, and m∈N. The conclusive iterative procedure for (35) that was obtained by applying the method [43] is shown below:

$$\begin{array}{c} \left\{ \begin{array}{c} h_{n+1}=h_{0}+\frac{\hbar^{\varsigma_{1}}}{\Gamma (\varsigma_{1}+1)}\sum_{\iota =0}^{n}\left({\left(1+n-\iota \right)}^{\varsigma_{1}}-{\left(n-\iota \right)}^{\varsigma_{1}}\right)G\left(t_{\iota },h\left(t_{\iota }\right)\right),\\ n=0,1\cdots ,m. \end{array}\right. \end{array}$$ (36)

Consequently, the Caputo NiV epidemic model (13) numerical solution is achieved as

$$\begin{array}{l} V_{n+1}=V_{0}+\frac{\hbar^{\varsigma_{1}}}{\Gamma (\varsigma_{1}+1)}\sum_{\iota =0}^{n}((1+n-\iota {)}^{\varsigma_{1}}-{\left(n-\iota \right)}^{\varsigma_{1}})(pI_{F_{\iota }}-\theta V_{\iota }),\\ S_{F_{n+1}}=S_{F_{0}}+\frac{\hbar^{\varsigma_{1}}}{\Gamma (\varsigma_{1}+1)}\sum_{\iota =0}^{n}((n-\iota +1{)}^{\varsigma_{1}}-{\left(n-\iota \right)}^{\varsigma_{1}})(\Pi_{F}-\frac{\beta_{1}S_{F_{\iota }}V_{\iota }}{N_{F_{\iota }}}-d_{F}S_{F_{\iota }}),\\ I_{F_{n+1}}=I_{F_{0}}+\frac{\hbar^{\varsigma_{1}}}{\Gamma (\varsigma_{1}+1)}\sum_{\iota =0}^{n}((1-\iota +n{)}^{\varsigma_{1}}-{\left(n-\iota \right)}^{\varsigma_{1}})(\frac{\beta_{1}S_{F_{\iota }}V_{\iota }}{N_{F_{\iota }}}-d_{F}I_{F_{\iota }}),\\ S_{H_{n+1}}=S_{H_{0}}+\frac{\hbar^{\varsigma_{1}}}{\Gamma (\varsigma_{1}+1)}\sum_{\iota =0}^{n}((n-\iota +1{)}^{\varsigma_{1}}-{\left(n-\iota \right)}^{\varsigma_{1}})\\ \;\times (\Pi_{H}-\frac{\left(\beta_{2}V_{\iota }+\beta_{3}I_{H_{\iota }}+\beta_{4}\kappa D_{H_{\iota }}\right)S_{H_{\iota }}}{N_{H_{\iota }}}-c_{1}S_{H_{\iota }}+\gamma R_{H_{\iota }}+\zeta V_{H_{\iota }}),\\ V_{H_{n+1}}=V_{H_{0}}+\frac{\hbar^{\varsigma_{1}}}{\Gamma (\varsigma_{1}+1)}\sum_{\iota =0}^{n}((1+n-\iota {)}^{\varsigma_{1}}-{\left(n-\iota \right)}^{\varsigma_{1}})\times (\xi S_{H_{\iota }}-c_{2}V_{H_{\iota }},),\\ I_{H_{n+1}}=I_{H_{0}}+\frac{\hbar^{\varsigma_{1}}}{\Gamma (\varsigma_{1}+1)}\sum_{\iota =0}^{n}((n+1-\iota {)}^{\varsigma_{1}}-{\left(n-\iota \right)}^{\varsigma_{1}})\\ \;\times (\frac{\left(\beta_{2}V_{\iota }+\beta_{3}I_{H_{\iota }}+\beta_{4}\kappa D_{H_{\iota }}\right)S_{H_{\iota }}}{N_{H_{\iota }}}-c_{3}I_{H_{\iota }}),\\ T_{H_{n+1}}=T_{H_{0}}+\frac{\hbar^{\varsigma_{1}}}{\Gamma (\varsigma_{1}+1)}\sum_{\iota =0}^{n}((n+1-\iota {)}^{\varsigma_{1}}-{\left(n-\iota \right)}^{\varsigma_{1}})\times (\rho I_{H_{\iota }}-c_{4}T_{H_{\iota }},),\\ R_{H_{n+1}}=R_{H_{0}}+\frac{\hbar^{\varsigma_{1}}}{\Gamma (\varsigma_{1}+1)}\sum_{\iota =0}^{n}((n-\iota +1{)}^{\varsigma_{1}}-{\left(n-\iota \right)}^{\varsigma_{1}})\times (\alpha_{1}I_{H_{\iota }}+\alpha_{2}T_{H_{\iota }}-c_{5}R_{H_{\iota }}),\\ D_{H_{n+1}}=D_{H_{0}}+\frac{\hbar^{\varsigma_{1}}}{\Gamma (\varsigma_{1}+1)}\sum_{\iota =0}^{n}((n+1-\iota {)}^{\varsigma_{1}}-{\left(n-\iota \right)}^{\varsigma_{1}})\times (d_{1}I_{H_{\iota }}-\nu_{H}D_{H_{\iota }}). \end{array}$$ (37)

### 6.1 Simulation and discussion

The numerical technique (37) and the parameter values listed in Table 1 are used to simulate the Caputo fractional model (13). In simulation, the time level is considered up to 200 days to better illustrate the time behavior of the model solution. The simulations are acquired for the following two scenarios, taking into account different values of *ς*_1_ ∈ (0, 1].

#### 6.1.1 The dynamics of the model for $R_{0}>1$

**Fig 7:**
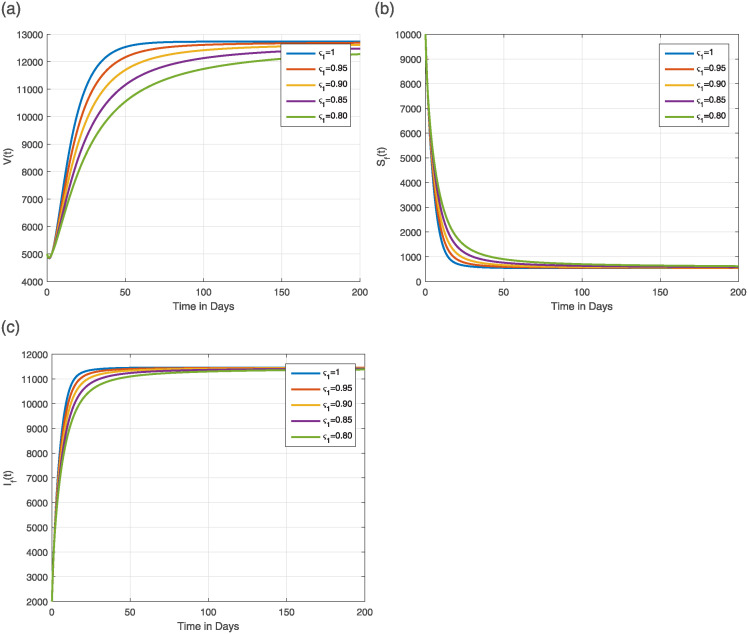
Simulation of (a) V(t), (b) $S_{F}\left(t\right)$, and (c) $I_{F}\left(t\right)$ classes in the Caputo-NiV computational model (13) with $\varsigma_{1}=0.80,0.85,0.90,0.95,1.0$. The parameters are tabulated in Table 1 and $R_{0}>1$.

**Fig 8:**
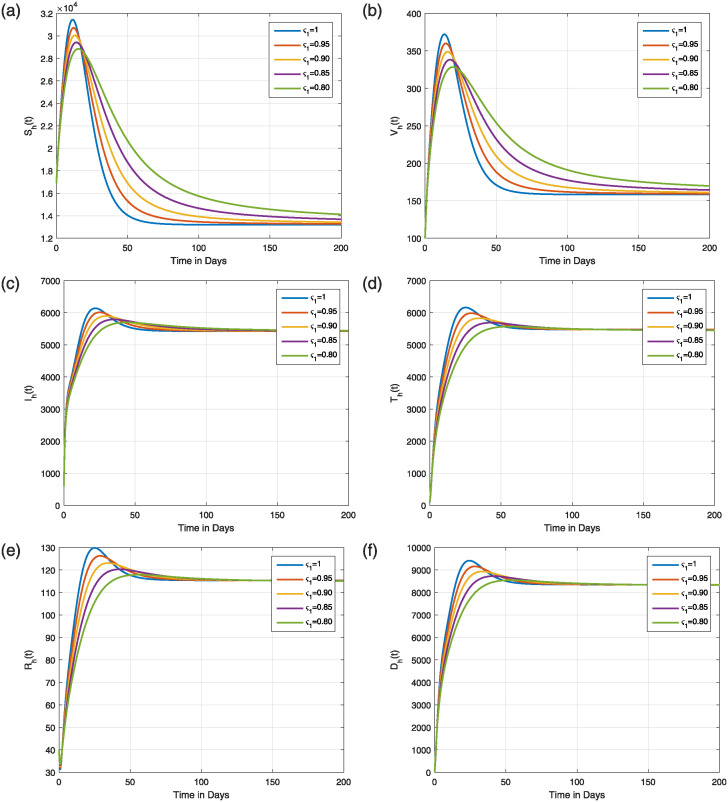
Dynamics of (a) susceptible (b) vaccinated (c) infectious (d) treated (e) recovered (f) deceased human classes in the model (13) with $\varsigma_{1}=0.80,0.85,0.90,0.95,1$ and when $R_{0}>1$.

In the present case, $R_{0}$ exceeds unity as utilizing the parameters of the baseline values, which are listed in Table 1. Fig 7(a)–7(c) illustrates the dynamical features of the class V(t), the various classes of flying foxes, for both classical (integer) and fractional values of *ς*_1_. The dynamics of every human population class are studied in Fig 8, with sub-Figs (a-f), respectively, for different values of *ς*_1_. Regardless of the parameters of *ς*_1_, it is clear that the solution of the model always approaches the NiV-endemic state. However, for a memoryless NiV model (*ς*_1_ = 1), the model reaches a stationary state quickly, within a short time and as the value of *ς*_1_ decreases, the time required to reach this stationary state increases.

#### 6.1.2 The dynamics of the model for $R_{0}<1$

**Fig 9:**
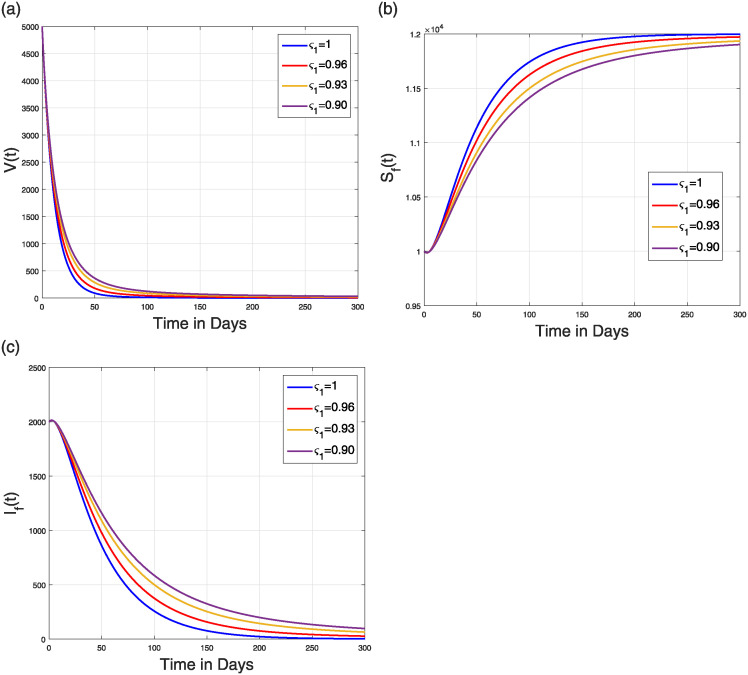
Simulation describing the dynamical features of (a) V(t), (b) $S_{F}\left(t\right)$, and (c) $I_{F}\left(t\right)$ in the model (13) with $\varsigma_{1}=0.80,0.85,0.90,0.95,1$ and when $R_{0}<1$.

**Fig 10:**
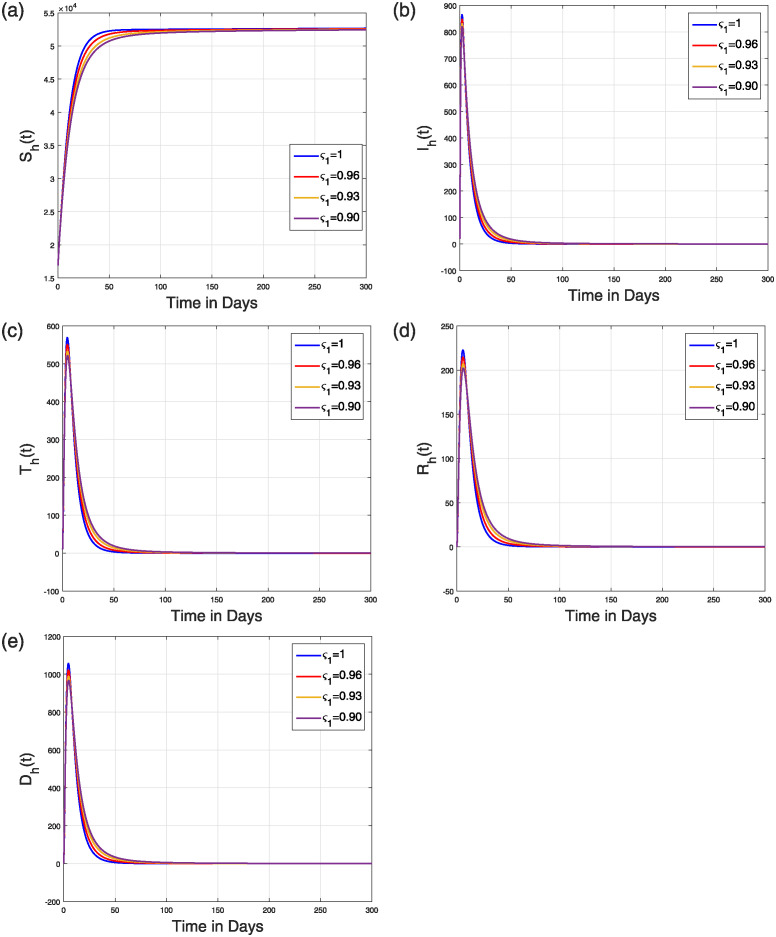
Simulation describing the dynamical features of (a) susceptible (b) infectious (c) treated (d) recovered (e) decease humans in the Caputo-NiV model (13) with $\varsigma_{1}=0.90,0.93,0.96,1$ and when $R_{0}<1$.

To ensure that $R_{0}$ is less than 1, we take into account the values of *β*_1_ = 0.250, *β*_2_ = 0.250, *β*_3_ = 0.150, *β*_3_ = 0.350, *d*_*F*_ = 0.25. The values of the remaining parameters are listed in Table 1. Fig 9(a)–9(c) displays the outcomes of the viral concentration, susceptible, and infected flying-fox population. Fig 10(a)–10(e) shows the simulation of the human population’s groups. In this case, the simulation showed that for all values of *ς*_1_, the NiV model converges to the NVFE. Therefore, by decreasing disease transmission rates and raising flying fox natural death rates, infection can be completely eradicated. Similar to the previous case, $\varsigma_{1}=1$, the model reaches NVFE quickly, within a short time, and as the value of *ς*_1_ decreases, the time required to reach this stationary state increases.

## 7 The extension of NiV epidemic FF model

We designed a more generic transmission model using an advanced modeling approach to better understand the crossover dynamics of the NiV. For this, the Caputo type operator for FF is utilized. $\varsigma_{2}$ and *ς*_1_ are the values assigned to the fractional and fractal dimensions, respectively. The FF NiV epidemic model is accomplished in the subsequent system.

DFF0,tς1,ς2(V(t))=pIF-θV,DFF0,tς1,ς2(SF(t))=ΠF-β1SFVNF-dFSF,DFF0,tς1,ς2(IF(t))=β1SFVNF-dFIF,DFF0,tς1,ς2(SH(t))=ΠH-(β2V+β3IH+β4κDH)SHNH-c1SH+γRH+ζVH,DFF0,tς1,ς2(VH(t))=ξSH-c2VH,DFF0,tς1,ς2(IH(t))=(β2V+β3IH+β4κDH)SHNH-c3IH,DFF0,tς1,ς2(TH(t))=ρIH-c4TH,DFF0,tς1,ς2(RH(t))=α1IH+α2TH-c5RH,DFF0,tς1,ς2(DH(t))=d1IH-νHDH. (38)

We now move forward to prove the fundamental characteristics of the FF model (38).

### 7.1 Uniqueness and existence of FF model

The fixed-point theory and the renowned Picard-Lindelöf theorem are used to establish the essential results of the NiV FF model (38). In order to achieve the required proof of the FF model (38) is changed as the next generic problem:

{DFF0,tς1,ς2h(t)=Q(t,h(t)),h(0)=h0,0<t<∞. (39)

The Cauchy problem (39) is integrated to give:

$$\begin{array}{c} \frac{1}{\Gamma (1-\varsigma_{1})}\frac{d}{dt}\int_{0}^{t}{\left(t-\varsigma \right)}^{-\varsigma_{1}}Q\left(t,h\left(t\right)\right)d\varsigma =\varsigma_{2}t^{\varsigma_{2}-1}Q\left(t,h\left(t\right)\right). \end{array}$$ (40)

Applying the integral after replacing the right-hand side by the Caputo operator yielded the formula shown in [46].

$$\begin{array}{c} h\left(t\right)=h\left(0\right)+\frac{\varsigma_{2}}{\Gamma (\varsigma_{1})}\int_{0}^{t}{\left(t-\varsigma \right)}^{\varsigma_{1}-1}\varsigma^{\varsigma_{2}-1}Q\left(\varsigma ,h\left(\varsigma \right)\right)d\varsigma . \end{array}$$ (41)

By utilizing the Picard-Lindelof theorem, we may formally define the following:

$$\prod_{c_{1}}^{c_{2}}=I_{m}\left(t_{m}\right)\times \bar{B_{0}\left(p_{0}\right)},$$

where,

$$I_{m}\left(t_{m}\right)=\left[t_{m-c_{1}},t_{m+c_{1}}\right],\;\bar{B_{0}\left(p_{0}\right)}=\left[c_{2}+t_{0},c_{2}+t_{0}\right].$$

Further, we define the following operator

$$\Pi .C\left[I_{n}\left(t_{m}\right),B_{c_{2}}\left(t_{m}\right)\right]\to C\left(I_{n}\left(t_{m}\right),A_{c_{2}}\left(t_{m}\right)\right),$$

where

$$\begin{array}{c} \Pi \varphi \left(t\right)=h\left(0\right)+\frac{\varsigma_{2}}{\Gamma (\varsigma_{1})}\int_{0}^{t}{\left(t-\varsigma \right)}^{\varsigma_{1}-1}\varsigma^{\varsigma_{2}-1}Q\left(\varsigma ,\varphi \left(\varsigma \right)\right)d\varsigma . \end{array}$$ (42)

Our forthcoming proof aims to show that the operator defined in Eq (42) maps a complete normed metric space onto itself. Moreover, it is necessary to demonstrate that the mapping satisfies the contraction criterion. During our preliminary phase, we showed that:

$$\begin{array}{c} \|\Pi \phi (t)-h(0)\|\leq c, \end{array}$$ (43)

where the norm is given by

$$\begin{array}{lll} \left\|\Pi \phi (t)-h(0)\right\| & \leq & \frac{\varsigma_{2}}{\Gamma (\varsigma_{1})}\int_{0}^{t}{\left(t-\varsigma \right)}^{\varsigma_{1}}\varsigma^{\varsigma_{2}-1}\|Q(\varsigma ,h(\varsigma )){\|}_{\infty }d\varsigma \\ & \leq & \frac{\varsigma_{2}}{\Gamma (\varsigma_{1})}H\int_{0}^{t}{\left(t-\varsigma \right)}^{\varsigma_{1}}\varsigma^{\varsigma_{2}-1}d\varsigma , \end{array}$$ (44)

$$H={\left\|Q\right\|}_{\infty },$$

where,

$${\left\|\Theta \right\|}_{\infty }=sup_{t\in \prod_{c_{1}}^{c_{2}}}\left\|\Theta \left(t\right)\right\|.$$

Furthermore, by employing the assumption *ς* = *t y*, we may get from the aforementioned integral.

$$\begin{array}{ccc} \left\|\Pi \phi (t)-h(0)\right\| & \leq & \frac{\varsigma_{2}H}{\Gamma (\varsigma_{1})}t^{\varsigma_{1}+\varsigma_{2}-1}B\left(\varsigma_{1},\varsigma_{2}\right), \end{array}$$ (45)

$$\begin{array}{c} \|\Pi \phi \left(t\right)-h\left(0\right)\|<c\Rightarrow H<\frac{c\Gamma (\varsigma_{1})}{\varsigma_{2}\left(a^{\varsigma_{2}+\varsigma_{1}-1}\right)B\left(\varsigma_{2},\varsigma_{1}\right)}. \end{array}$$ (46)

Considering *ϕ*_1_, *ϕ*_2_ $\in C[I_{n}\left(t_{m}\right),B_{c_{2}}\left(t_{m}\right)]$, we reached to the subsequent result

$$\begin{array}{lll} \left\|\Pi \phi_{1}-\Pi \phi_{2}\right\| & \leq & \left(\frac{\varsigma_{2}H}{\Gamma \left(\varsigma_{1}\right)}t^{\varsigma_{2}+\varsigma_{1}-1}B\left(\varsigma_{1},\varsigma_{2}\right)\right)\|\phi_{1}-\phi_{2}\|\\ & < & \left(\frac{\varsigma_{2}L}{\Gamma \left(\varsigma_{1}\right)}a^{\varsigma_{2}+\varsigma_{1}-1}B\left(\varsigma_{1},\varsigma_{2}\right)\right)\|\phi_{1}-\phi_{2}\|. \end{array}$$ (47)

Finally, if the following criteria are fulfilled, we validate the property of contraction based on the previous analysis.

$$\begin{array}{c} H<\frac{\Gamma (\varsigma_{1})}{\varsigma_{2}\left(a^{\varsigma_{2}+\varsigma_{1}-1}\right)B\left(\varsigma_{1},\varsigma_{2}\right)}. \end{array}$$ (48)

### 7.2 Computational study for NiV model in FF case

We provide a computational scheme for the FF model shown in (38) in this section. The iterative strategy for the FF model is developed based on the reference [46]. Using the RL operator, the NiV FF may be rewritten as follows:

$$\begin{array}{c} \frac{1}{\Gamma (1-\varsigma_{1})}\frac{d}{dt}\int_{0}^{t}{\left(t-\varsigma \right)}^{-\varsigma_{1}}h\left(\varsigma \right)d\varsigma \frac{1}{\varsigma_{2}t^{\varsigma_{2}-1}}. \end{array}$$ (49)

Consequently, the problem (39) can be described as

DRL0,tς1(h(t))=ς2tς2-1[Q(t,h(t))]. (50)

Additionally, we substitute the Caputo-type for the RL derivative in order to make use of the integer ICs. Consequently,

$$\begin{array}{ccc} h(t) & = & h\left(0\right)+\frac{\varsigma_{2}}{\Gamma (\varsigma_{1})}\int_{0}^{t}\varsigma^{\varsigma_{2}-1}{\left(t-\varsigma \right)}^{\varsigma_{1}-1}Q\left(\varsigma ,h\left(\varsigma \right)\right)d\varsigma . \end{array}$$ (51)

By setting *t* = *t*_*n*+1_, in (51), we have

$$\begin{array}{ll} h^{n+1} & =h_{0}+\frac{\varsigma_{2}}{\Gamma \left(\varsigma_{1}\right)}\int_{0}^{t_{n+1}}\varsigma^{\varsigma_{2}-1}{\left(t_{n+1}-\varsigma \right)}^{\varsigma_{1}-1}Q\left(\varsigma ,h\left(\varsigma \right)\right)d\varsigma ,\\ & =h_{0}+\frac{\varsigma_{2}}{\Gamma \left(\varsigma_{1}\right)}\overset{n}{\underset{v=0}{\sum }}\int_{t_{v}}^{t_{v+1}}\varsigma^{\varsigma_{2}-1}{\left(t_{n+1}-\varsigma \right)}^{\varsigma_{1}-1}Q\left(\varsigma ,h\left(\varsigma \right)\right)d\varsigma . \end{array}$$ (52)

Furthermore, the approximation of Q(ς,h(ς)) in (52) is determined by the interpolation of Lagrangian across the time interval $\left[t_{j},t_{j+1}\right]$:

$$\begin{array}{ccc} Q\left(\varsigma ,h\left(\varsigma \right)\right)\approx h_{j}\left(\varsigma \right) & = & \frac{\varsigma -t_{j-1}}{t_{j}-t_{j-1}}t_{j}^{\varsigma_{2}-1}Q\left(t_{j},h\left(t_{j}\right)\right)-\frac{\varsigma -t_{j}}{t_{j}-t_{j-1}}t_{j-1}^{\varsigma_{2}-1}Q\left(t_{j-1},h\left(t_{j-1}\right)\right). \end{array}$$ (53)

Following the approximation in (53), the below iterative formula is obtained.

$$\begin{array}{ccc} h^{n+1}\left(t\right) & = & h_{0}+\frac{\varsigma_{2}}{\Gamma (\varsigma_{1})}\sum_{j=0}^{n}\int_{t_{j}}^{t_{j+1}}\lambda^{\varsigma_{2}-1}{\left(t_{n+1}-\varsigma \right)}^{\varsigma_{1}-1}h_{j}\left(\varsigma \right)d\varsigma . \end{array}$$ (54)

Finally, the solution of (54) provides the following iterative formulae

$$\begin{array}{ll} h^{n+1} & =h_{0}+\frac{\varsigma_{2}\hbar^{\varsigma_{1}}}{\Gamma \left(\varsigma_{1}+2\right)}\overset{n}{\underset{j=0}{\sum }}[t_{j}^{\varsigma_{2}-1}Q\left(t_{j},h\left(t_{j}\right)\right)\\ & \times \left({\left(n+1-j\right)}^{\varsigma_{1}}\left(2+n+\varsigma_{1}-j\right)-{\left(n-j\right)}^{\varsigma_{1}}\left(n-j+2+2\varsigma_{1}\right)\right)\\ & -t_{j-1}^{\varsigma_{2}-1}Q\left(t_{j-1},h\left(t_{j-1}\right)\right)\times \left({\left(n+1-j\right)}^{\varsigma_{1}+1}-{\left(n-j\right)}^{\varsigma_{1}}\left(n+1-j+\varsigma_{1}\right)\right)]. \end{array}$$ (55)

### 7.3 Simulation of FF model

We use the NiV FF model (38) to simulate the disease outbreak for various values of fractal *ς*_2_ ∈ (0, 1] and fractional *ς*_1_ ∈ (0, 1] dimensions. The iterative scheme (55) is successfully used to simulate three different cases. We considered the parameter baseline values from Table 1 and the initial conditions as in the fractional model. We present the outcomes in graphical form and discuss their implications in the following sections.

**Fig 11:**
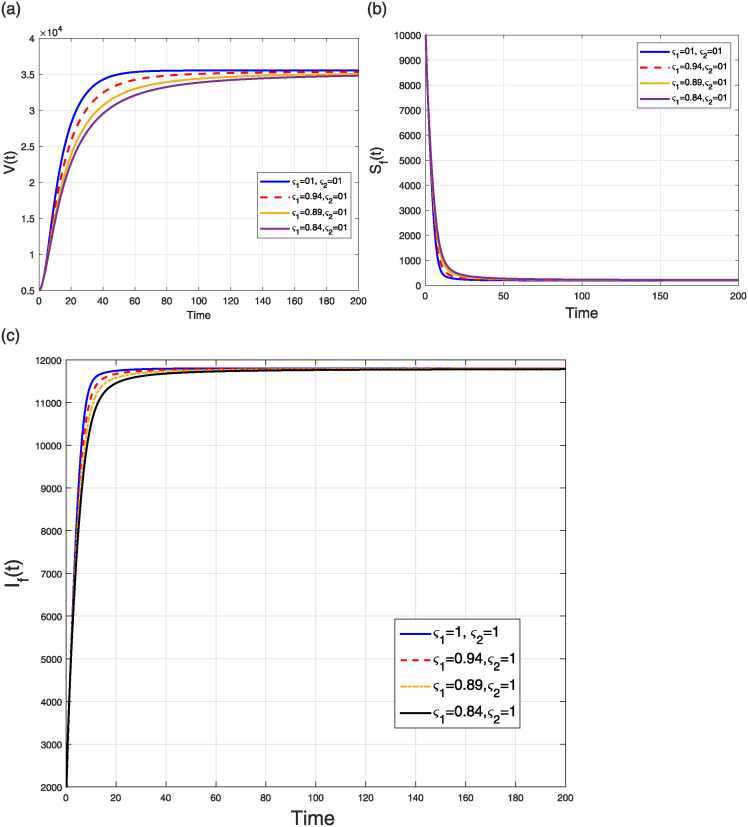
Simulation of (a) V(t), (b) $S_{F}\left(t\right)$, and (c) $I_{F}\left(t\right)$ classes in the FF model (38) when *ς*_1_ = 0.84, 0.89, 0.94, 1.00 and *ς*_2_ = 1.

**Fig 12:**
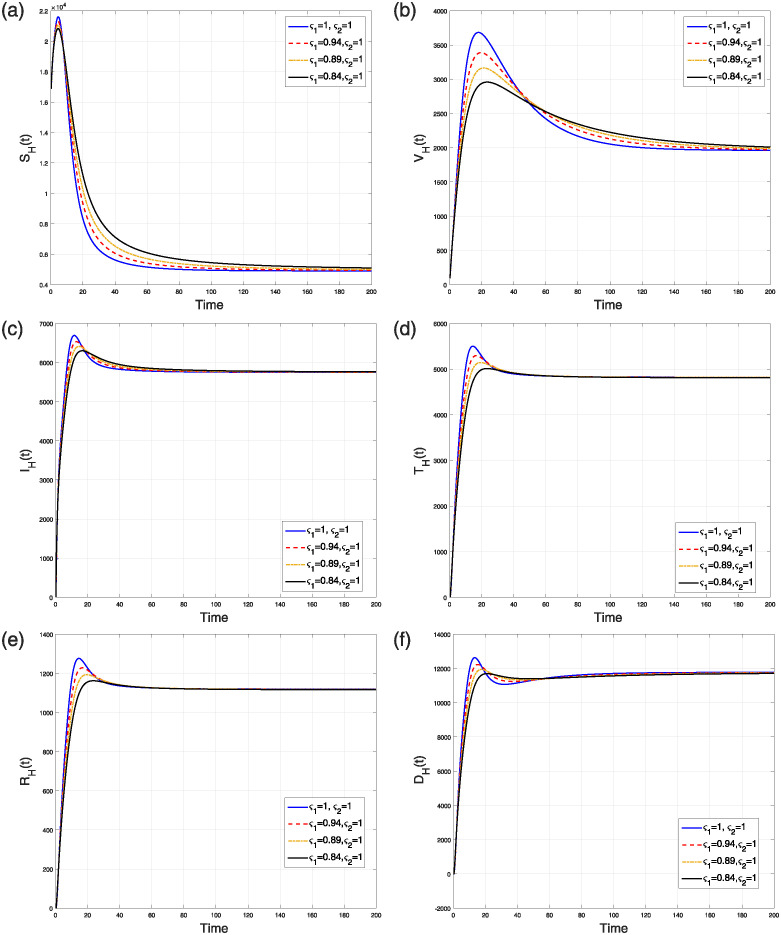
Case 1: Simulation of (a) susceptible (b) vaccinated (c) infectious (d) treated (e) recovered (f) deceased humans compartments in the FF model (38) when *ς*_1_ = 0.84, 0.89, 0.94, 1.00 and *ς*_2_ = 1.

The first scenario aims to simulate the NiV compartmental model (38) by considering the fractal to the integer case *ς*_2_ = 1 and a varying fractional parameter *ς*_1_. We study the impacts of the fractional parameter only, throughout the range *ς*_1_ ∈ (0, 1], spanning four distinct fractional orders. The numerical results are illustrated in two separate figures, specifically Figs 11(a)–11(c) and 12(a)–12(f). Fig 11(a)–11(c) depicts the numerical results regarding the levels of viral concentration and the number of individuals in the flying fox classes. Fig 12(a)–12(f) illustrates the dynamics of different human population groups. The fractal and fractional dimension phenomena of the model’s curves converge to the endemic state.

**Fig 13:**
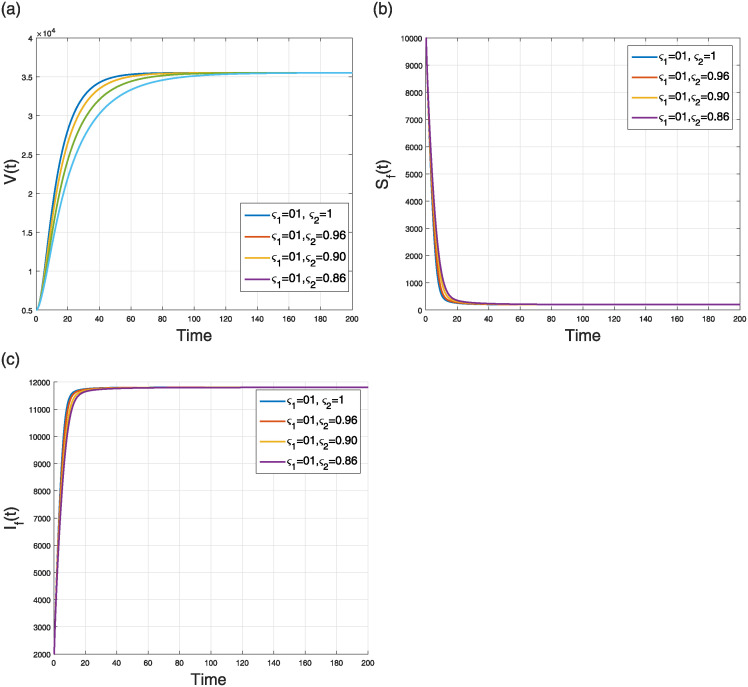
Simulation of (a) V(t), (b) $S_{F}\left(t\right)$, and (c) $I_{F}\left(t\right)$ classes in the NiV FF transmission model (38) when *ς*_1_ = 0.86, 0.90, 0.96, 1.000 and *ς*_2_ = 1.

**Fig 14:**
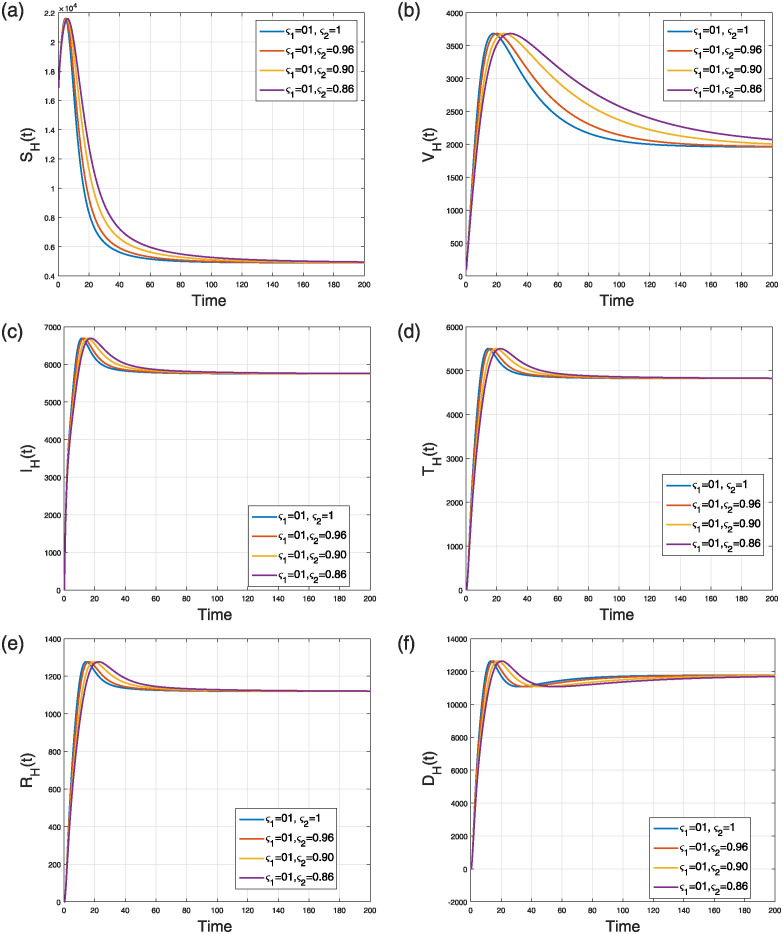
Simulation of (a) susceptible (b) vaccinated (c) infectious (d) treated (e) recovered (f) deceased humans compartments in the NiV FF transmission model (38) when *ς*_2_ = 0.86, 0.90, 0.96, 1.0 and *ς*_1_ = 1.

This scenario explains the impact of varying the fractal order only while keeping the fractional operator fixed at an integer value (i.e., *ς*_2_ = 1) on the dynamics of the model (38). The simulation was conducted using four distinct fractal orders, with *ς*_1_ varying within the range (0, 1]. The analysis of several model populations’ simulations is presented in Figs 13(a)–13(c) and 14(a)–14(f). Fig 13(a)–13(c) depicts the time behavior of the *V*(*t*), *S*_*F*_(*t*), and *I*_*F*_(*t*) compartments, respectively, whereas Fig 14(a)–14(f) analyze the time behavior of the susceptible, vaccinated, infectious, treated, recovered, and deceased individuals, respectively. Despite of the specific values of the fractional and fractal parameters, the solution curves converge to the stable state.

**Fig 15:**
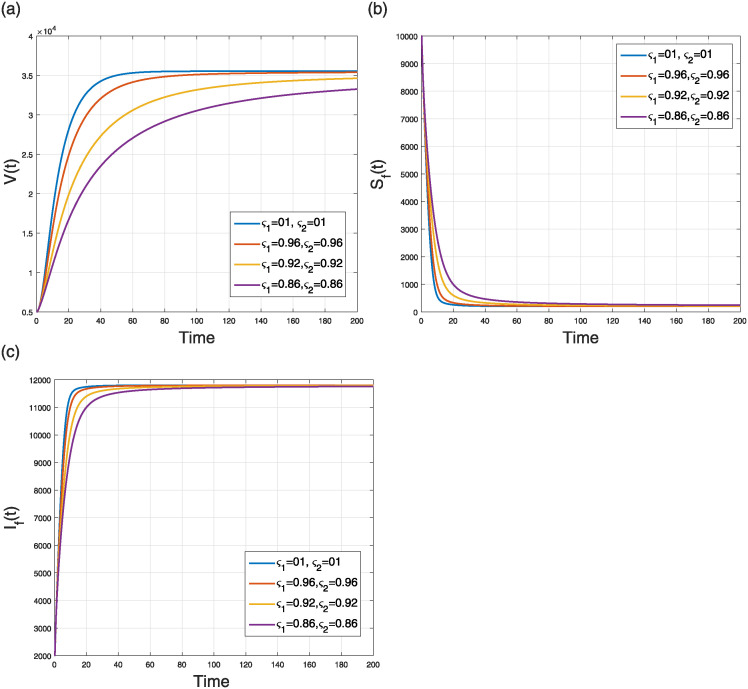
(a) V(t), (b) $S_{F}\left(t\right)$, and (c) $I_{F}\left(t\right)$ classes in the NiV FF transmission model (38) when *ς*_2_ = 0.86, 0.92, 0.96, 1.0 and *ς*_1_ = 0.86, 0.92, 0.96, 1.0.

**Fig 16:**
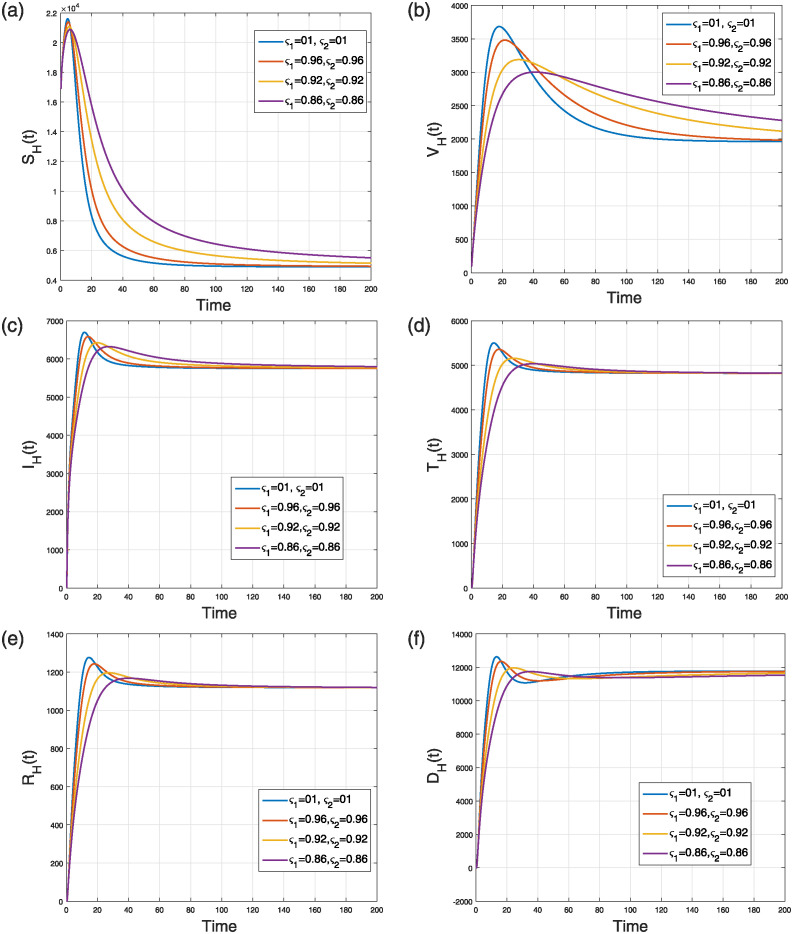
Simulation of (a) susceptible (b) vaccinated (c) infectious (d) treated (e) recovered (f) deceased human compartments in the FF transmission model (38) when *ς*_2_ = 0.86, 0.92, 0.96, 1.0 and *ς*_1_ = 0.86, 0.92, 0.96, 1.0.

Finally, the simulation of the FF epidemic model (38) is conducted by simultaneously varying both the fractional and fractal dimensions. The assigned values for both *ς*_1_ and *ς*_2_ are 0.86, 0.92, 0.96, and 1.0. The graphical interpretation of the results is examined in Figs 15(a)–15(c) and 16(a)–16(f). For smaller values of the fractional and fractal dimensions, the densities of the *V*(*t*) and *I*_*F*_(*t*) classes decrease, while the number of susceptible flying foxes increases. Overall, it is observed that for smaller values of both the fractal and fractional orders, the solution curves in all groups converge to the NiV endemic steady state over a longer period. This indicates that lower fractal and fractional dimensions lead to a slower approach to equilibrium, reflecting the influence of memory and complex dynamics on the epidemic’s progression.

In conclusion, across all simulated scenarios, the solution curves consistently approach the endemic stable state, demonstrating the model’s stable behavior. However, with the simultaneous non-integer values of *ς*_1_ and *ς*_2_, the system takes a longer time to attain equilibrium. The stable behavior of the FF epidemic model (38) at the NiV-free steady state can be verified for the parameters considered in the fractional epidemic model (13). Ultimately, it can be concluded that compartmental models incorporating FF operators provide a more comprehensive understanding of disease dynamics and enhance disease control strategies.

## 8 Conclusion

The present study investigated the behavior of NiV using an innovative computational modeling technique that integrates fractional and fractal-fractional operators. Human-to-human and food-borne viral transmissions were incorporated into the model formulation. In the modeling procedure, we employed a widely recognized Caputo-type derivative for both fractional and fractal-fractional scenarios. Most of the model parameters were calculated using NiV outbreaks and their clinical facts in Bangladesh. Using the fixed point and Picard-Lindelöf techniques, the conditions for the existence and uniqueness of both NiV epidemic models were demonstrated. In addition, we determined the model’s threshold number and all potential equilibria. The stability of the Caputo model was further investigated by employing the well-established Hyers-Ulam and Hyers-Rassias-Ulam stability criteria. Moreover, the models were solved using efficient numerical methods and extensive simulation results were conducted for various values of fractional order only and for combined fractal as well as fractional parameters. Graphical representations of global dynamics are depicted when the value of $R_{0}<1$ and $R_{0}>1$, indicating the global stable behavior of the solution curves toward the steady states. The results of this study showed that the NiV epidemic model with fractal-fractional Caputo operators consistently produces biologically realistic outcomes. These findings are expected to be valuable for health officials in their efforts to control the spread of the disease.
